# Unappreciated diversification of stem archosaurs during the Middle Triassic predated the dominance of dinosaurs

**DOI:** 10.1186/s12862-016-0761-6

**Published:** 2016-09-15

**Authors:** Christian Foth, Martín D. Ezcurra, Roland B. Sookias, Stephen L. Brusatte, Richard J. Butler

**Affiliations:** 1 Department of Geosciences, University of Fribourg/Freiburg, Fribourg, Switzerland; 2 SNSB, Bayerische Staatssammlung für Paläontologie und Geologie, München, Germany; 3 Department of Earth and Environmental Sciences and GeoBio-Center, Ludwig-Maximilians-Universität, München, Germany; 4 CONICET, Sección Paleontología de Vertebrados, Museo Argentino de Ciencias Naturales, Buenos Aires, Argentina; 5 School of Geography, Earth and Environmental Sciences, University of Birmingham, Birmingham, UK; 6 School of GeoSciences, University of Edinburgh, Edinburgh, UK

**Keywords:** Archosauromorpha, Archosauria, Cranial disparity, Geometric morphometrics, Evolution, Triassic

## Abstract

**Background:**

Archosauromorpha originated in the middle–late Permian, radiated during the Triassic, and gave rise to the crown group Archosauria, a highly successful clade of reptiles in terrestrial ecosystems over the last 250 million years. However, scientific attention has mainly focused on the diversification of archosaurs, while their stem lineage (i.e. non-archosaurian archosauromorphs) has often been overlooked in discussions of the evolutionary success of Archosauria. Here, we analyse the cranial disparity of late Permian to Early Jurassic archosauromorphs and make comparisons between non-archosaurian archosauromorphs and archosaurs (including Pseudosuchia and Ornithodira) on the basis of two-dimensional geometric morphometrics.

**Results:**

Our analysis recovers previously unappreciated high morphological disparity for non-archosaurian archosauromorphs, especially during the Middle Triassic, which abruptly declined during the early Late Triassic (Carnian). By contrast, cranial disparity of archosaurs increased from the Middle Triassic into the Late Triassic, declined during the end-Triassic extinction, but re-expanded towards the end of the Early Jurassic.

**Conclusions:**

Our study indicates that non-archosaurian archosauromorphs were highly diverse components of terrestrial ecosystems prior to the major radiation of archosaurs, including dinosaurs, while disparity patterns of the Ladinian and Carnian indicate a gradual faunal replacement of stem archosaurs by the crown group, including a short interval of partial overlap in morphospace during the Ladinian.

**Electronic supplementary material:**

The online version of this article (doi:10.1186/s12862-016-0761-6) contains supplementary material, which is available to authorized users.

## Background

**Fig. 1:**
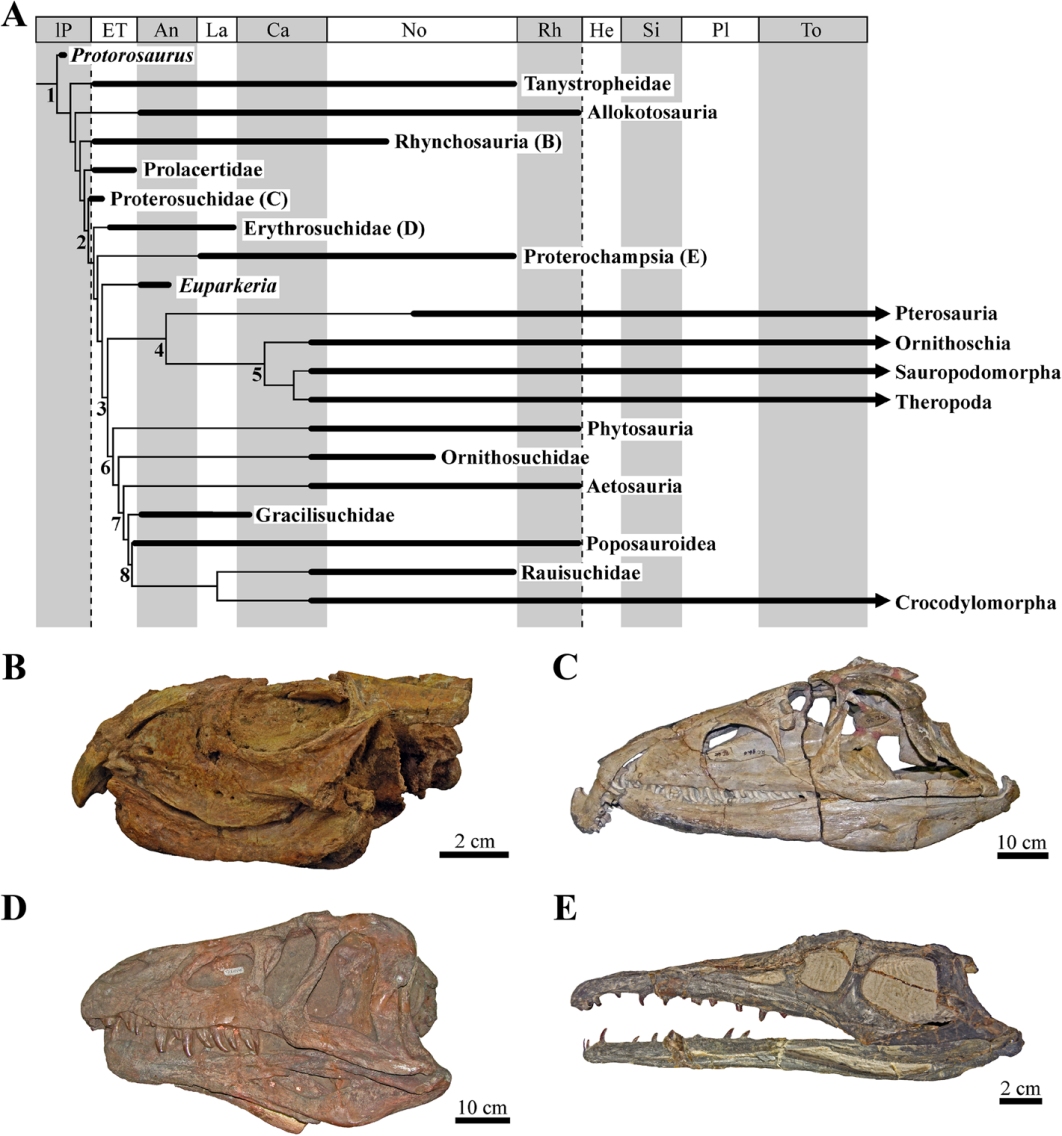
Archosauromorph relationships and skulls (in lateral view) exemplifying shape diversity. **a** Simplified time-calibrated phylogeny showing the main groups of archosauromorphs existing from the late Permian to the Early Jurassic. **b** The rhynchosaur *Bentonyx sidensis*. **c** The proterosuchid *Proterosuchus fergusi*. **d** The erythrosuchid *Erythrosuchus africanus*. **e** The proterochampsid *Gualosuchus reigi*

Living birds and crocodylians, as well as their extinct relatives including pterosaurs and non-avian dinosaurs, comprise the extraordinarily diverse and successful crown clade Archosauria. Archosauria in turn is part of a broader group, Archosauromorpha, which also includes a range of Permian and Triassic species that lie along the archosaur stem lineage, being more closely related to modern archosaurs than to lizards and snakes (lepidosaurs) [1]. Archosauromorphs originated in the middle–late Permian [2], but it was only after the end-Permian mass extinction (c. 252 Ma) that the group radiated spectacularly (Fig. 1a). They diversified through the 50 million years of the Triassic to dominate large-bodied niches on land and replace the previously successful synapsids [3], and gave rise to the earliest crown archosaurs by the late Early Triassic and the first dinosaurs by the Middle–early Late Triassic [4–9].

Scientific attention has mainly focused on the diversification of crown archosaurs (particularly dinosaurs: [4–9]), and non-crown-group taxa (i.e. non-archosaurian archosauromorphs) have been often overlooked in discussions of this key evolutionary event, although taxa such as *Proterosuchus* and *Euparkeria* were important in shaping our understanding of archosaur evolution [8, 9]. However, non-archosaurian archosauromorphs (i.e. taxa on the stem lineage leading towards archosaurs) formed a species rich component of Triassic ecosystems (>90 valid species) and achieved high morphological diversity (Fig. 1b-e), including highly specialised herbivores (e.g. [10, 11]), large apex predators (e.g. [12, 13]), marine predators with extremely elongated necks (e.g. [14]), armoured crocodile-like forms (e.g. [15, 16]), and possibly even turtles (e.g. [17–19]). Although the currently available fossils of Permian archosauromorphs are limited to only four species, phylogenies imply that all the main lineages of non-archosauriform archosauromorphs extend back into the Permian, indicating high, but incompletely sampled, taxonomic diversity [2], (Fig. 1a). The few known Permian archosauromorph body fossils and footprints reveal that the group had already achieved a broad geographic distribution and locomotor differentiation, including sprawling and erect postures [20]. As a result, a comprehensive understanding of the Triassic rise of archosaurs must be framed within the context of this broader radiation of closely related stem-taxa.

Previous work attempting to quantify the morphological diversification of archosauromorphs during the Triassic has focused solely on crown archosaurs [4, 21, 22], and has primarily used discrete characters derived from cladistic data matrices assembled for phylogenetic analyses [4, 22]. The utility of such datasets for quantifying ecological variation is, however, debated [23]. Here we attempt for the first time to quantify patterns of cranial morphological diversity during the first 100 million years of the early archosauromorph radiation using an alternative approach that has great power to capture morphological variation - geometric morphometrics. We compare morphospace occupation and temporal changes in disparity in non-archosaurian archosauromorphs versus archosaurs, and provide new insights into this important diversification event.

## Methods

### Taxonomic sampling and geometric morphometrics

To explore the morphological variation of the archosauromorph skull (excluding the lower jaw) we sampled 73 late Permian–Early Jurassic species represented by published skull reconstructions of adult (or advanced subadult) individuals in lateral view (see Additional file 1: Table S1). Here, skull reconstructions were carefully evaluated qualitatively with respect to completeness and degree of taphonomic damage and deformation of the original fossil material (based on photographs or first-hand observations), in order to minimize the impact of these factors on shape analyses [24]. As a consequence, reconstructions relying on very incomplete or strongly deformed material were not taken into account.

We used two-dimensional geometric morphometrics to study variation in cranial morphology. Cranial shape was captured with 15 landmarks and 38 semi-landmarks, using the software tpsDig2 [25]. The landmarks used in this study were classified as either type 1 (given point defined along the articulation between two bones) or type 2 (points of maximum curvature and extremities) [26], while the shapes of cranial openings and the overall skull outline were captured by semi-landmarks, which were plotted at equal intervals along the curves of the structures they were defining [26, 27] (see Additional file 1: Figure S1, Table S4). The resulting dataset was analysed in MorphoJ [28] and superimposed using Generalized Procrustes Analyses (GPA) [29] (see Additional file 2). Although the positions of semi-landmarks depend on the position of other landmarks, and, therefore, contain less shape information, we treated landmarks and semi-landmarks as equivalent for GPA [30], because in some species the sliding process creates considerable artificial deformation on the Procrustes shape [31]. The Procrustes residuals were then converted into a covariance matrix and subjected to Principal Component Analysis (PCA), also in MorphoJ (Additional file 2). This method transforms the variation among individuals into new sets of independent variables (principal components) that are linear combinations of the original set, with zero covariance. Because the principal components (PCs) describe successively smaller amounts of total variation of the sample, it becomes possible to describe a large proportion of variation using a small number of variables [30, 32]. The PCs define a morphospace that depicts the overall spread of variation among taxa. The scores for the taxa on the PCs summarise the skull shape of each taxon, and therefore are shape proxies that can be used in macroevolutionary analyses to quantify major trends in skull evolution.

### Disparity analyses, phylogeny and time calibration

We used the principal component (PC) scores to calculate temporal and phylogenetic variation in morphological disparity [33, 34]. Temporal disparity curves were calculated for two main groupings of archosauromorphs: the paraphyletic assemblage of non-archosaurian archosauromorphs; and crown group Archosauria. Because recent cladistic analyses find differing placements for phytosaurs, a major group of long-snouted semi-aquatic Triassic archosauromorphs, we constructed two datasets: one in which phytosaurs are included within crown Archosauria [1, 5, 35, 36] and the other in which they are treated as non-archosaurian archosauromorphs [8]. However, our preferred hypothesis is that they are crown archosaurs based upon the most extensive phylogenetic analysis of early archosauromorphs conducted to date [36]. Within crown Archosauria, we also compiled disparity curves for the two main subgroups: Pseudosuchia (crocodile line archosaurs) and Ornithodira (dinosaurs and their close relatives). The measures for pseudosuchians were calculated both including and excluding phytosaurs.

As noted above, non-archosaurian archosauromorphs represent a paraphyletic assemblage, and the same is true for crown group Archosauria in our analysis, because all Middle Jurassic–Recent archosaurs are excluded from our disparity calculations. However, quantification of morphospace occupation for a composite non-archosaurian Archosauromorpha allows us to assess the amount of morphological variation shown by the archosaur stem group, how this changed through time, and the relative importance of the crown group in determining the overall pattern of archosauromorph disparity. Unfortunately, sample sizes are too small for us to estimate disparity patterns for individual non-archosaurian archosauromorph clades, such as Erythrosuchidae or Proterochampsidae.

To increase the resolution of the disparity analyses, and partially fill in gaps in the fossil record where archosauromorphs are poorly sampled but must have been more diverse based on phylogenetic ghost lineages, we also included PC scores of hypothetical ancestors in the disparity analyses [37]. We assembled two informal, time-calibrated supertrees based on recent literature, which differ only in the position of phytosaurs (see Additional file 1: Tables S5, S6; Additional file 3). To reconstruct reliable ancestral shapes for those clades whose origin falls into the final time bin of our analysis (Toarcian), we added 38 additional archosauromorph species from younger time intervals (Middle Jurassic–Late Cretaceous) to the supertree and morphometric dataset (see Additional file 1: Tables S2-S3). In addition, a sensitivity analysis was conducted without hypothetical ancestors to estimate the impact of the latter on the results (see Additional file 1: Tables S18-S23, Figure S6).

Time calibration of the trees was based on the ages of terminal taxa (see Additional file 1: Tables S1-S3), and a minimum branch length of 0.1 Myr was used, which essentially takes the fossil record at face value. This approach ensures that inferred hypothetical ancestors will always be placed conservatively in the youngest bin they could possibly occupy.

To predict ancestral morphologies, the PC scores of the terminal taxa were mapped as continuous characters onto the topologies in Mesquite 3.04 [38], using squared change parsimony [39], and ancestral node values were optimized. The resulting ancestral PC scores were then added to the overall dataset of PC scores of the terminal taxa.

For the temporal disparity curves, taxa were binned into approximately equal time intervals spanning the late Permian to Toarcian. Taxa from the Wuchiapingian and Changhsingian (late Permian) and Induan and Olenekian (Early Triassic) were combined into single bins, whereas the Norian was split into early and late Norian bins. Terminal taxa were binned according their midpoint-age (i.e., the midpoint of the range of uncertainty of their age), while hypothetical ancestors were binned based on the age of time-calibration. As a result of using the midpoint-age for terminal taxa, we assign skull shapes only to one time bin, although the total range of occurrence of some taxa may span over more than one bin, usually as a result of stratigraphic uncertainty rather than genuine stratigraphic range. Our methodology does not at present deal with situations where a clade (e.g. Erythrosuchidae) is present and sampled in two time bins, but is currently unsampled in a third intervening time bin although it must have been present (so-called ‘Lazarus taxa’). Solving this problem would require a more sophisticated approach involving evolutionary modelling of character changes that goes beyond the scope of this paper; in any case, the number of instances of Lazarus taxa in our dataset is likely to be small given the relatively restricted time frame and number of time bins.

Between-group disparity comparisons were made between non-archosaurian archosauromorphs and crown archosaurs, and between pseudosuchians and ornithodirans, within individual time bins.

For all disparity calculations, we used sum of variances (SoV) as our disparity metric, as it is more robust to sample size differences than other metrics [33, 40]. Sum of variances was estimated for each group in every bin using R 3.1.2 [41]. Here, the minimum number of taxa for each group per bin was set at three. Bins with fewer taxa were not considered. All calculations are based on the first eight PC scores (83.2 % of total shape variation), which summarize the significant shape variation within the whole dataset, based on the broken stick method [42] performed in PAST [43].

Differences in disparity between adjacent time bins and between different groups within a single bin were tested for significance in R using a permutation test with 10,000 replicates, which takes into account the sample size difference between the two comparisons [44]. This procedure tests whether a certain group had more or less total disparity than another by keeping the sample size of each group constant and shuffling the taxa randomly between the groups. Furthermore, we also tested whether two groups have significantly overlapping or different positions in morphospace using nonparametric multivariate analysis of variance (NPMANOVA) in PAST, which tests for significant differences in the distribution of groups in morphospace on the basis of permutation [45]. All permutations were conducted using the first eight PC scores, which were transformed into a Euclidean distance matrix, permuted with 10,000 replications. Comparisons were made using the Bonferroni correction, to reduce the likelihood of type 1 statistical errors given the large number of comparisons performed.

## Results

### Major cranial shape variation

**Fig. 2:**
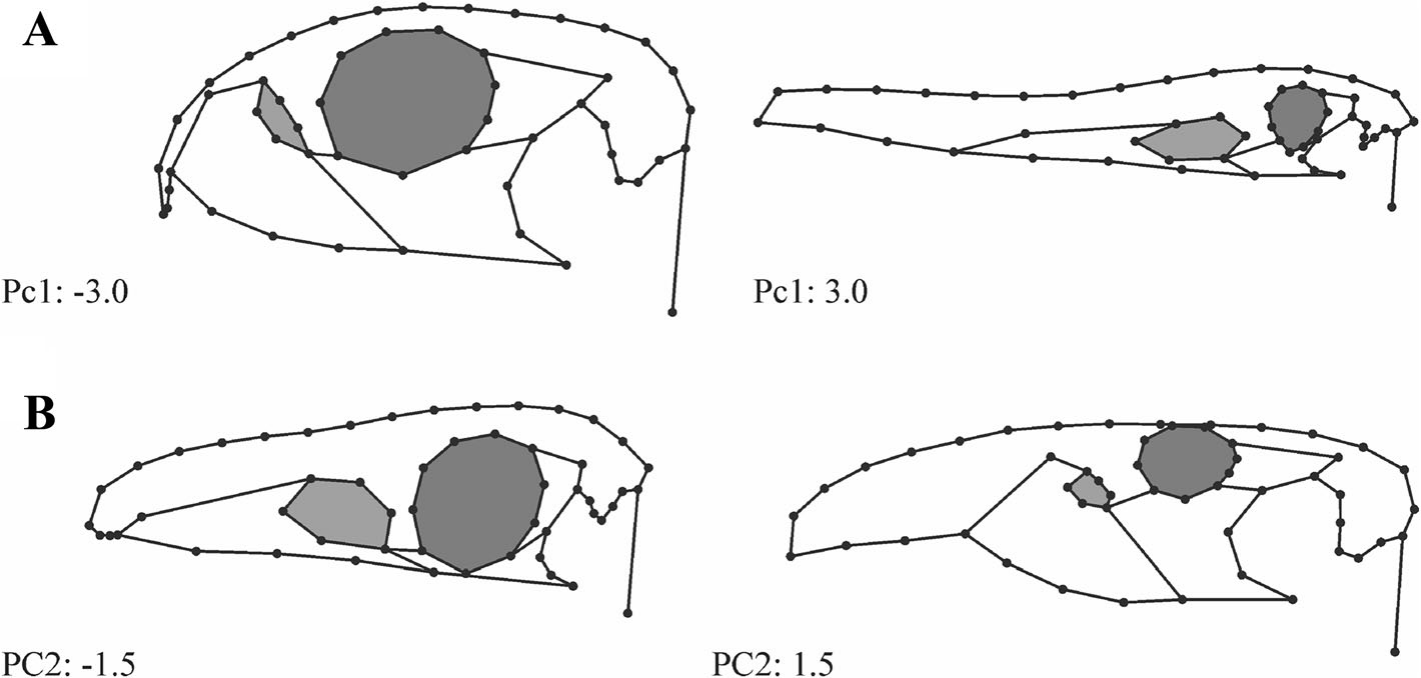
Main shape variation of archosauromorphs living from the Permian to the end of the Early Jurassic. **a** First principal component. **b** Second principal component. The antorbital fenestra is coloured in *light grey*, the orbit is coloured in *dark grey*

The majority of cranial shape variation is captured by the first two axes of the PCA (percentages of total variance: PC1 = 42.1 %; PC2 = 12.8 %). PC1 describes the fundamental ways in which early archosauromorph skulls differ from each other: the anteroposterior and dorsoventral proportions of the skull, mainly affected by the extension of the anterior end and inclination of the premaxilla, the depths of the maxilla, the size of the jugal and postorbital, and the anteroposterior and dorsoventral dimensions of the orbital and postorbital regions. This variation further affects the overall curvature of the skull roof, the anteroposterior length and position of the antorbital fenestra, the size and anterodorsal extension of the orbit, the dorsoventral height of the infratemporal fenestra, and the position of the jaw joint along an anterodorsal-posteroventral axis (Fig. 2). The second PC describes the extension of the posterior end of the premaxilla, relative proportion of depth and length of the maxilla, relative size and extension of the anteroventral margin of the antorbital fenestra, relative size and extension of the posteroventral margin of the orbit, height of the skull roof in the post-rostral region in relation to the snout, relative depth of the jugal and postorbital, relative length of the squamosal, extension of the anterior margin of the infratemporal fenestra, and the position of the jaw joint in an anterodorsal-posteroventral direction (Fig. 2).

### Morphological disparity of Archosauromorpha during the Permian–Early Jurassic

**Fig. 3:**
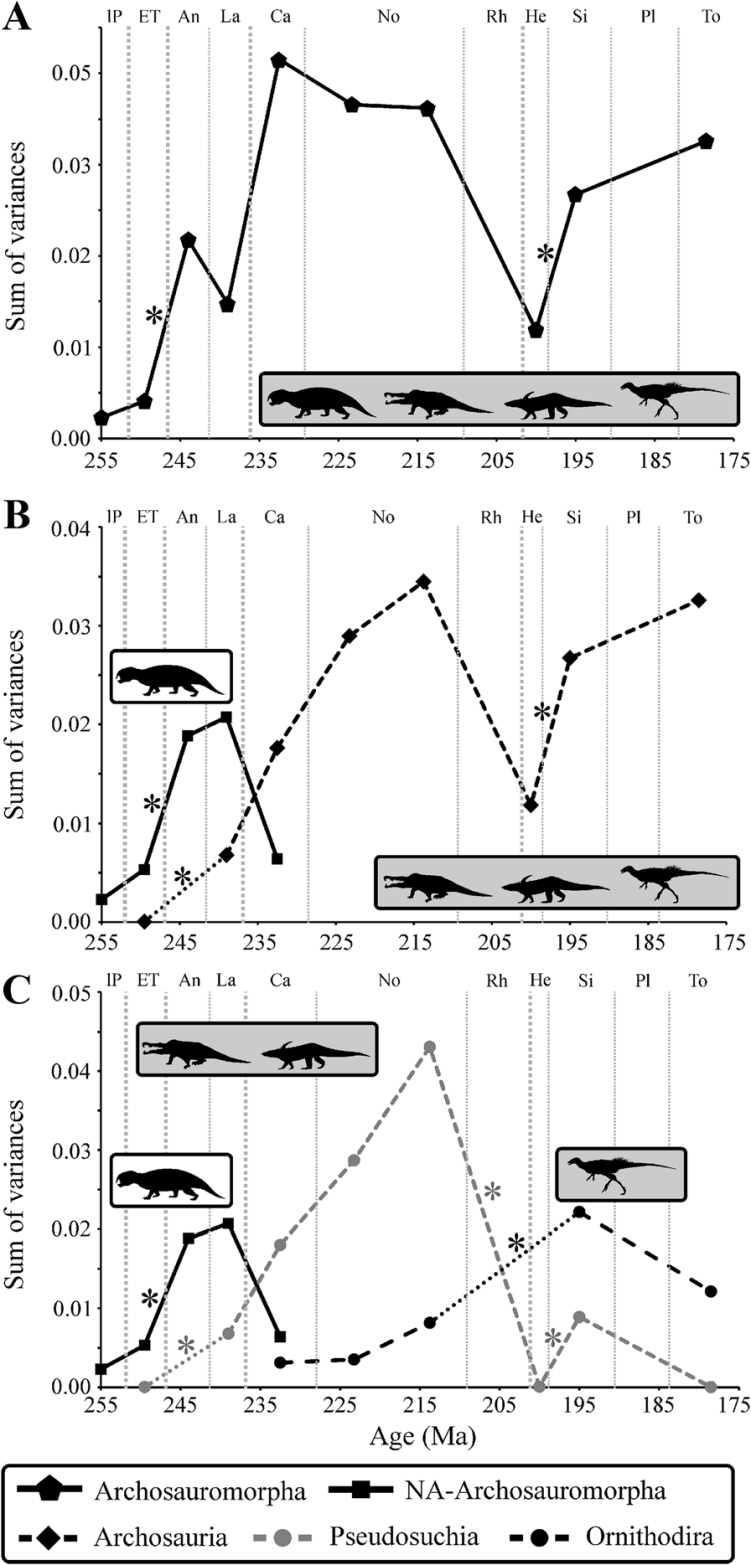
**a** Temporal variation of disparity for all archosauromorphs through time from the late Permian to the late Early Jurassic. **b** Temporal pattern of non-archosaurian archosauromorphs (*solid line* with *black squares*) and crown archosaurs (*dashed line* with *black diamonds*) when phytosaurs are members of the crown. **c** Temporal pattern of non-archosaurian archosauromorphs (*solid line* with *black squares*), pseudosuchians (*dashed line* with *grey circles*), and ornithodirans (*dashed line* with *black circles*). Significant changes between subsequent time bins are marked with an *asterisk. Lines* with tiny dots mark missing time bins due to small sample sizes. All silhouettes taken from (www.phylopic.org)

The cranial disparity of all archosauromorphs sampled increases from the late Permian to the Anisian, with a significant difference between the Early Triassic and the Anisian (Fig. 3a). In the Ladinian a small, non-significant decrease occurs, before disparity increases again in the Carnian to reach the overall maximum within the studied time span. In the Norian, disparity is comparable to that of the Carnian, being only slightly (non-significantly) lower. Disparity declines strongly in the Hettangian, and subsequently increases again until the Toarcian, with a significant difference between the Hettangian and Sinemurian (Fig. 3a, see Additional file 1: Tables S7, S8). Based on the NPMANOVA results, the archosauromorph cranium exhibits significant shifts of morphospace from the late Permian to the Early Triassic, from the Early Triassic to the Anisian, and from the Sinemurian to the Toarcian (see Additional file 1: Table S8).

### Morphological disparity of non-archosaurian Archosauromorpha and Archosauria

When phytosaurs are treated as members of crown Archosauria (Ezcurra 2016) [36], the cranial disparity of non-archosaurian archosauromorphs increases from the late Permian to the Ladinian, with significant differences from the Early Triassic to the Anisian (Fig. 3b). After reaching a maximum in the Ladinian, non-archosaurian archosauromorph cranial disparity decreases in the Carnian (the last bin sampled for the group), with the last non-archosaurian archosauromorphs occurring in the Rhaetian [46]. The first record of Archosauria is found in the Early Triassic [47] and from this point their cranial disparity increases continuously until the late Norian, followed by a decline in the Hettangian and a significant re-expansion until the Toarcian. The post-Hettangian increase includes a significant change from the Hettangian to the Sinemurian. The cranial disparity of non-archosaurian archosauromorphs is significantly higher than that of archosaurs (including phytosaurs) in the Early and Middle Triassic, whereas archosaurs possess a higher disparity in the Carnian, although the latter is not significant (Fig. 3a, see Additional file 1: Tables S7, S9, S13).

**Fig. 4:**
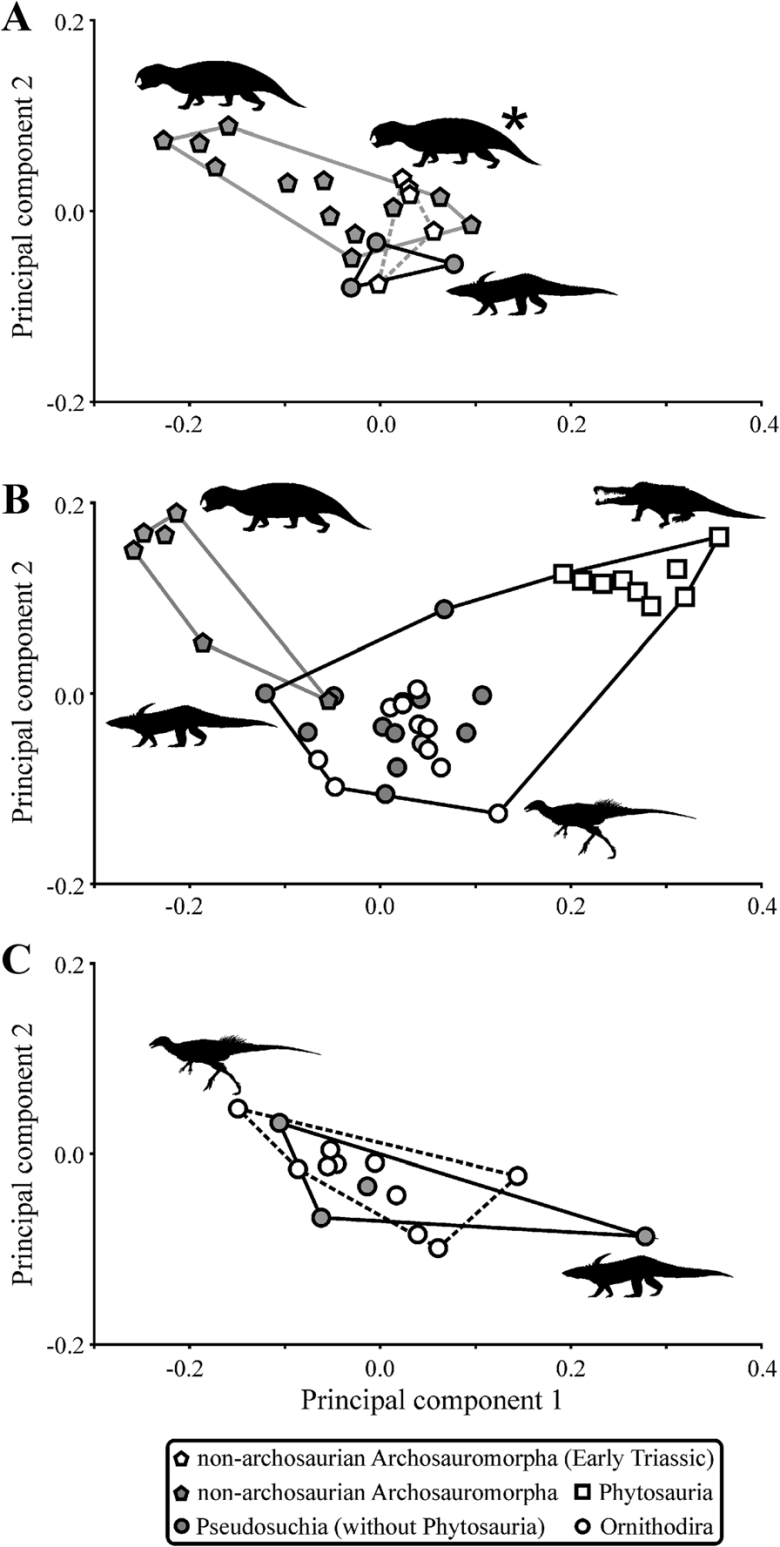
Morphospace occupation of archosauromorphs. **a** Two-dimensional morphospace of all Early (*grey dashed line* with *white pentagons*, silhouette marked with *asterisk*) and Middle Triassic non-archosaurian archosauromorphs (*grey solid line* with *grey pentagons*) and pseudosuchians (*black solid line* with *grey circles*). **b** Two-dimensional morphospace of Late Triassic archosauromorphs showing the morphospace of non-archosaurian archosauromorphs (*grey solid line* with *grey pentagons*) and crown archosaurs (*black solid line*) with pseudosuchians (without phytosaurs) (*grey circles*), phytosaurs (*white squares*), and ornithodirans (*white circles*). **c** Two-dimensional morphospace of all Early Jurassic archosaurs with ornithodirans (*black dashed line* with *white circles*) and pseudosuchians (*black solid line* with *grey circles*). Note that graphs show the morphospace of entire epochs and are not always equivalent to the time bins. All silhouettes taken from (www.phylopic.org)

Based on the NPMANOVA, non-archosaurian archosauromorphs exhibit significant shifts of morphospace from the late Permian to the Early Triassic, from the Early Triassic to the Anisian and from the Ladinian to the Carnian, while in Archosauria significant shifts occur from the late Norian to the Hettangian and from the Sinemurian to the Toarcian. When compared to each other, non-archosaurian Archosauromorpha and Archosauria occupy significantly different areas within morphospace during the Early Triassic and the Carnian, but are not significantly separated from each other in the Ladinian (only two Anisian archosaurs are sampled and differences in morphospace with non-archosaurian archosauromorphs cannot be tested statistically) (Fig. 4, see Additional file 1: S9, S13, S16). Results showing the cranial disparity and morphospace occupation when phytosaurs are sister group of crown-archosaurs and when hypothetical ancestors are not included are described in the Additional files (see Additional file 1: Tables S7, S9, S13, S17; S18-S23, Figure S6).

Within crown archosaurs, cranial disparity of pseudosuchians increases from the Early Triassic to the late Norian, with a significant difference between the Early Triassic and the Ladinian. After reaching a maximum in the late Norian, disparity declines in the Hettangian, and then increases in the Sinemurian (both significantly), followed by a further decrease in the Toarcian (Fig. 3, see Additional file 1: Tables S7, S14). By contrast, cranial disparity of ornithodirans increases from the Carnian continuously until the Sinemurian, with a significant change from the late Norian to the Sinemurian (only one Hettangian ornithodiran is sampled and differences in morphospace cannot be tested statistically). In the Toarcian, cranial disparity decreases again (Fig. 3, see Additional file 1: Tables S7, S15).

The NPMANOVA indicates that the increase from the Ladinian to the Carnian and the decline after the end-Triassic extinction in Pseudosuchia resulted in a significant shift in morphospace (see Additional file 1: Table S14). By contrast, Ornithodira show a significant shift from the Carnian to the early Norian (see Additional file 1: Table S15). When compared to each other, Pseudosuchia (including phytosaurs) and Ornithodira occupy significantly different areas in morphospace over the entire sampled time span (Additional file 1: Table S16).

The overall disparity trends described here remain valid when hypothetical ancestors were excluded from the data set, although the temporal resolution was less precise (see Additional file 1: Tables S18-S23, Figure S6).

## Discussion

### Comparisons with previous analyses of early archosaur disparity and limitations of results

There are several similarities between our results and previous studies that were based primarily on discrete characters. Brusatte et al. [4] recovered pseudosuchian disparity considerably higher than that of dinosaurs during the entire Late Triassic, similarly to our analysis. By contrast, Avemetatarsalia (=ornithodirans in our analyses) had similar morphological diversity to Pseudosuchia during the Norian [48], which differs from our results. However, this could be an artefact because the current study focuses on cranial shape only, whereas Brusatte et al. [48] took the entire skeletal anatomy into account. The high disparity of ornithodirans during the Norian in the analysis of Brusatte et al. [48] probably results from the inclusion of pterosaurs, which underwent strong postcranial morphological modifications during their early evolution [49].

Across the Triassic–Jurassic (TJ) boundary, Brusatte et al. [48] identified a decline in pseudosuchian disparity, as recovered here. However, Brusatte et al. [48] recovered static ornithodiran and dinosaur disparity across the TJ boundary, and argued that this was inconsistent with an opportunistic replacement of pseudosuchians by ornithodirans/dinosaurs. By contrast, we recover significant increases in both ornithodiran and dinosaur disparity through the same time interval, which is potentially consistent with opportunistic replacement scenarios. Our results are also in agreement with those of Stubbs et al. [21], who found a decline of disparity in the mandibular shape of pseudosuchians across the TJ boundary. By contrast, Toljagic and Butler [22], who investigated morphological variation in pseudosuchians on the basis of discrete characters of the entire skeleton, found that their disparity did not change significantly across the TJ boundary due to an earliest Jurassic adaptive radiation of crocodylomorphs that balanced disparity loss due to the extinction of non-crocodylomorph pseudosuchians.

Several previous studies have found converging signals when different disparity proxies were applied [50–52], while the current study reveals partial differences between disparity analyses using discrete characters [22, 48] and those based on geometric morphometric data. These discrepancies could be due to a variety of reasons, including differences in taxonomic sampling, weaknesses of either the discrete character or morphometric datasets, or discrepancies between cranial and postcranial disparity in highly specialized clades such as pterosaurs. Future research is needed to better understand why different approaches to estimating disparity change do not always produce consistent results.

One further issue deserves comment. The disparity results from our morphometric study could potentially be affected by differences in the sample sizes of groups in each time bin. Because we use sum of variances as our disparity metric (see above), this influence should be minimized compared to other disparity metrics that can be more strongly affected by sample size ([33, 40]). Additionally, correlation tests show that most disparity curves are not significantly correlated with sample size over time (Additional file 1: Table S24). Only for Pseudosuchia did we find a significant correlation between disparity through time and sample size. However, rather than being artefactual, this correlation could be explained instead by a parallel increase of species diversity and cranial disparity in the early phase of the pseudosuchian radiation during the Triassic, resulting in the evolution of very different ecomorphotypes with distinct skull shapes [5, 8]. By contrast, due to the extinction of many of these specialized pseudosuchians at the end of the Triassic [8], but the survival of crocodylomorphs with relatively generalized skull shapes, we see another correlation between low sample size and cranial disparity during the Jurassic. Thus, we suggest that the significant correlations found here do not reflect a dependence of cranial disparity on sample size, but a true evolutionary signal.

### Macroevolutionary implications

Macroevolutionary analyses that have explored the faunal changes that occurred in vertebrate assemblages during the Triassic have focused mainly on comparisons of taxic diversity and morphological disparity between the two archosaur lineages (i.e. Pseudosuchia and Ornithodira) or between dinosaurs and other terrestrial tetrapods [4, 6, 53–55]. Thus, these analyses were mainly restricted to the Middle–Late Triassic and mostly ignored the role of non-archosaurian archosauromorphs in these evolutionary events. Our analysis found previously unappreciated high morphological disparity for non-archosaurian archosauromorph skulls with a major peak during the Middle Triassic. These results indicate that non-archosaurian archosauromorphs were highly diverse components of terrestrial ecosystems in terms of skull shape prior to the major diversification of crown archosaurs, including dinosaurs. The high cranial disparity of non-archosaurian archosauromorphs during the Middle Triassic was the result of sustained increases from the late Permian to the Ladinian. In particular, the significant changes in non-archosaurian archosauromorph morphospace occupation between the late Permian, Early Triassic and Anisian, and the significant disparity increase from the Early Triassic to Anisian indicate a shift from a rather morphologically homogenous archosauromorph assemblage immediately after the end-Permian mass extinction, mainly dominated by proterosuchid-like forms, to a more disparate one by the Middle Triassic [56]. This change may be related to the stabilization of ecosystems and opening of new ecological niches following an interval of global instability [57, 58].

After the Middle Triassic peak, the cranial disparity of non-archosaurian archosauromorphs (when phytosaurs are included within Archosauria) decreased abruptly during the Carnian. This decrease is non-significant, but it is larger in absolute values than the increases documented during the Early–Middle Triassic. Beyond this decrease, a significant change in morphospace occupation occurs between the Ladinian and Carnian. This change may indicate a taxonomically selective extinction event. These results further indicate that the cranial disparity of numerous non-archosaurian archosauromorph clades was in decline before they disappeared in the Norian and end-Triassic extinction events [53]. Interestingly, during the Ladinian the morphospace occupation of non-archosaurian archosauromorphs does not differ significantly from that of archosaurs, but the former are significantly more disparate than the latter. By contrast, in the Carnian the morphospace occupations of archosaurs and non-archosaurian archosauromorphs differ significantly from each other, with archosaurs being more disparate than non-archosaurian archosauromorphs. This could be the result of the extinction of Middle Triassic non-archosaurian taxa which had overlapping morphospace with that of Late Triassic archosaurs. This change restricted the morphospace of non-archosaurian archosauromorphs that survived into the Norian to ecologically specialized groups, such as hyperodapedontine rhynchosaurs, trilophosaurids, tanystropheids, and proterochampsids.

The disparity patterns documented here are consistent with a gradual faunal replacement event, with a short interval of partial morphospace overlap between non-archosaurian archosauromorphs and archosaurs during the Ladinian, followed by decline of the former group and expansion of the morphospace of the latter. The macroevolutionary events described above change if phytosaurs are considered to be outside of Archosauria (see Additional file 1). Nevertheless, even if phytosaurs are not crown archosaurs, the overall evolutionary patterns found here (including the Middle Triassic non-archosaurian archosauromorph disparity peak) are still valid for the group composed of non-archosaurian archosauromorphs to the exclusion of phytosaurs and other archosaurs.

During the Late Triassic and Early Jurassic the cranial disparity of archosaurs increased and surpassed that previously reached by non-archosaurian archosauromorphs. Prior to the end-Triassic mass extinction, pseudosuchians comprised the majority of archosaur disparity, whereas disparity was considerably, but non-significantly, lower in ornithodirans. By contrast, the re-expansion of archosaur disparity in the Early Jurassic stems from the diversification of ornithodirans, the disparity of which surpasses that of pseudosuchians after the end-Triassic extinction (Fig. 3, see Additional file 1: Figure S5, Tables S7, S14, S15).

## Conclusions

Based on cranial shape analyses of archosauromorph reptiles spanning from the Late Permian to the Early Jurassic, we demonstrate that the success of archosaurs (crocodylians, dinosaurs, birds) in Jurassic to modern ecosystems was predated by unappreciated high skull shape diversity in stem archosaurs during the Middle Triassic. This high early cranial diversity reflects very disparate cranial morphologies, which are potentially related to different environmental adaptations and feeding ecologies. A series of possible replacement and extinction events between the Middle and Late Triassic resulted in a decline of non-archosaurian archosauromorph disparity and taxonomic diversity, while the ecomorphological diversification of crown archosaurs, which began in the Middle Triassic (predominately pseudosuchians) and continued after the end-Triassic mass extinction (predominately ornithodirans, including dinosaurs), went hand-in-hand with an increase in cranial disparity.

## Additional files

Additional file 1: Additional information on taxon sampling, landmark positions, phylogeny, discussion points, tables and figures. (PDF 494 kb) Additional file 2: MorphoJ data set. (TXT 10497 kb) Additional file 3: Supertree topology with phytosaurs as crown group and stem group archosaurs. (NEX 20 kb)
