# Supplementary material for: Unappreciated diversification of stem archosaurs during the Middle Triassic predated the dominance of dinosaurs
Source: BMC Evol Biol. 2016 Sep 15;16:188. doi: 10.1186/s12862-016-0761-6 (PMC5024528; doi:10.1186/s12862-016-0761-6)
Supplement: Additional file 1: — Additional information on taxon sampling, landmark positions, phylogeny, discussion points, tables and figures. (PDF 494 kb) [file 12862_2016_761_MOESM1_ESM.pdf]

Additional file 1

Unappreciated diversification of stem archosaurs during the Middle Triassic predated the dominance of dinosaurs

Christian Foth[1,2,3], Martin D. Ezcurra[4,5], Roland B. Sookias[5], Stephen L. Brusatte[6], Richard J. Butler[5]

[1]Department of Geosciences, University of Fribourg/Freiburg, Fribourg, Switzerland

[2]SNSB, Bayerische Staatssammlung für Paläontologie und Geologie, München, Germany

[3]Department of Earth and Environmental Sciences and GeoBio-Center, Ludwig-Maximilians-Universität, München, Germany

[4]CONICET, Sección Paleontología de Vertebrados, Museo Argentino de Ciencias Naturales, Buenos Aires, Argentina

[5]School of Geography, Earth and Environmental Sciences, University of Birmingham, Birmingham, United Kingdom

[6]School of GeoSciences, University of Edinburgh, Grant Institute, James Hutton Road, Edinburgh EH9 3FE, UK

Content

1. **Taxon sampling**

   Table S1. List of Permian, Triassic and Early Jurassic taxa.

   Table S2. List of additional taxa from the Middle and Late Jurassic and Cretaceous used to calculate ancestral shapes.

   Table S3. Additional taxa for time calibration with data of occurrences.

2. **Description of landmarks and semi-landmarks**

   Table S4. Description of landmarks and semi-landmark description.

   Fig. S1. Distribution of landmarks and semi-landmarks.

3. **Phylogeny**

   Table S5. Sources for phylogenetic position of taxa included in the informal supertree.

   Table S6. Strength of the phylogenetic signal of the skull shape of Archosauromorpha.

   Fig. S2. Supertree topology mapped into the morphospace of archosauromorphs.

4. **Disparity and NPMANOVA results**

   Table S7. Sum of variances of Archosauromorpha through time.

   Table S8. Statistical differences of sum of variances and morphospace position between subsequent time bins for all Archosauromorpha.

   Table S9. Statistical differences of sum of variances and morphospace position between subsequent time bins for non-archosaurian Archosauromorpha.

   Table S10. Sum of variances of non-archosaurifom Archosauromorpha and non-archosaurian Archosauriformes through time.

   Table S11. Statistical differences of sum of variances and morphospace position between subsequent time bins for non-archosaurifom Archosauromorpha and non-archosaurian Archosauriformes when phytosaurs are members of the crown-group of Archosauria.

   Table S12. Statistical differences of sum of variances and morphospace position between subsequent time bins for non-archosaurifom Archosauromorpha and non-archosaurian Archosauriformes when phytosaurs are members of the stem-line of Archosauria.

Table S13. Statistical differences of sum of variances and morphospace position between subsequent time bins for Archosauria.

Table S14. Statistical differences of sum of variances and morphospace position between subsequent time bins for Pseudosuchia.

Table S15. Statistical differences of sum of variances and morphospace position between subsequent time bins for Ornithodira/Dinosauria.

Table S16. Statistical differences of sum of variances and morphospace position between different groups within time bin when phytosaurs are crown-archosaurs.

Table S17. Statistical differences of sum of variances and morphospace position between different groups within time bin when phytosaurs are stem-archosaurs.

5. Additional results and discussion points

5.1. General disparity trend within archosauromorph cranium

5.2. The effect of the phylogenetic position of Phytosauria

Fig. S3. Disparity of archosauromorphs through time from the late Permian to the end of the Early Jurassic with phytosaurs as members of the stem group.

Fig. S4. Two-dimensional morphospace of Late Triassic archosaurmorphs showing the morphospace of non-archosaurian archosauromorphs including phytosaurs and crown archosaurs.

5.3. Cranial disparity trends within Ornithodira

Fig. S5. Temporal disparity of pseudosuchians and dinosaurs from the Carnian to the end of the Early Jurassic.

5.4. The impact of ancestral shapes for disparity analyses

Table S18. Sum of variances of Archosauromorpha (just terminal taxa) through time.

Table S19. Statistical differences of sum of variances and morphospace position between subsequent time bins for all Archosauromorpha (just terminal taxa).

Table S20. Statistical differences of sum of variances and morphospace position between subsequent time bins for non-archosaurian Archosauromorpha (just terminal taxa).

Table S21. Statistical differences of sum of variances and morphospace position between subsequent time bins for Archosauria (just terminal taxa).

Table S22. Statistical differences of sum of variances and morphospace position between subsequent time bins for Pseudosuchia (just terminal taxa).

Table S23. Statistical differences of sum of variances and morphospace position between subsequent time bins for Ornithodira/Dinosauria (just terminal taxa).

Fig. S6. Comparison of disparity through time for various archosauromorph groups, if shapes of hypothetical ancestors were included or excluded.

5.5. The impact sample size for disparity analyses

Table S24. Results of ordinary least squares (OLS) and generalised least squares (GLS) regression of skull shape disparity sample size per bin.

5.6. The age of the Chañares Formation

6. References

1. Taxon sampling

Table S1. List of Permian, Triassic and Early Jurassic taxa used in the present analyses with data of occurrences (in million of years) and sources of skull reconstructions. The stratigraphic ages of the terminal taxa, upon which the time-calibration is based, were taken from the Paleobiology Database (www.paleobiodb.org), but checked for accuracy and modified if required.

Species	Clade	Age	Time	Source
Tanystropheus longobardicus	'Protorosauria'	Anisian-Ladinian	242	Nosotti 2007
Langobardisaurus pandolfii	'Protorosauria'	late Norian	213.1	Saller et al. 2013
Protorosaurus speneri	'Protorosauria'	middle Wuchiapingian	257.3	Gottmann-Quesada and Sander 2009
Prolacerta broomi	Prolacertidae	Induan-early Olenekian	250.7	Ezcurra and Butler 2015a
Proterosuchus fergusi	Proterosuchidae	Induan-early Olenekian	250.7	Ezcurra and Butler 2015b
Proterosuchus alexanderi	Proterosuchidae	Induan-early Olenekian	250.7	Ezcurra and Butler 2015b
Proterosuchus goweri	Proterosuchidae	Induan-early Olenekian	250.7	Ezcurra and Butler 2015b
Trilophosaurus buettneri	Allokotosauria	Norian	217	Sues and Fraser 2010
Azendohsaurus madagaskarensis	Allokotosauria	Ladinian-earliest Carnian	237	Flynn et al. 2010
Bentonyx sidensis	Rhynchosauria	late Anisian	243.3	Hone and Benton 2008
Hyperodapedon gordoni	Rhynchosauria	late Carnian-earliest Norian	228.95	Benton 1983
Hyperodapedon huxleyi	Rhynchosauria	late Carnian-earliest Norian	228.95	Chatterjee 1974
Hyperodapedon sanjuanensis	Rhynchosauria	late Carnian-earliest Norian	228.95	Langer and Schultz 2000
Mesosuchus browni	Rhynchosauria	early Anisian	245.9	Dilkes 1998
Rhynchosaurus articeps	Rhynchosauria	Anisian	244.6	Benton 1990
Rhynchosaurus brodiei	Rhynchosauria	Anisian	244.6	Benton 1990

Teyumbaita sulcognathus	Rhynchosauria	early Norian	222.45	Montefeltro et al. 2010
Erythrosuchus africanus	Erythrosuchidae	early Anisian	245.9	Nesbitt 2011
Garjainia prima	Erythrosuchidae	late Olenekian	248.2	Ezcurra and Butler 2015a
Shansisuchus shansisuchus	Erythrosuchidae	late Anisian	243.3	Wang et al. 2013
Chanaresuchus bonapartei	Proterochampsidae	Ladinian-earliest Carnian	237	Nesbitt 2011
Gualosuchus reigi	Proterochampsidae	Ladinian-earliest Carnian	237	Romer 1971
Euparkeria capensis	Euparkeriidae	early Anisian	245.9	Nesbitt 2011
Parasuchus hislopi	Phytosauria	late Carnian-earliest Norian	228.95	Sereno 1991
Smilosuchus gregorii	Phytosauria	early Norian	222.35	Nesbitt 2011
Brachysuchus megalodon	Phytosauria	late Carnian-early Norian	224.85	Case 1929
Mystriosuchus planirostris	Phytosauria	late Norian	213.1	Hungerbuhler 2002
Mystriosuchus westphali	Phytosauria	late Norian	213.1	Hungerbuhler 2002
Leptosuchus adamanensis	Phytosauria	Norian	217.75	Sues and Fraser 2010
Leptosuchus studeri	Phytosauria	early Norian	222.35	Case and White 1934
Nicrosaurus kapffi	Phytosauria	late Norian	213.1	Westphal 1976
Pseudopalatus pristinus	Phytosauria	late Norian-Rhaetian	209.5	Camp 1930
Riojasuchus tenuisceps	Ornithosuchidae	middle Norian	216	Nesbitt 2011
Ornithosuchus longidens	Ornithosuchidae	late Carnian-earliest Norian	228.95	Sereno 1991
Gracilisuchus stipanicicorum	Suchia	Ladinian-earliest Carnian	237	Nesbitt 2011
Qianosuchus mixtus	Poposauroidea	Anisian	244.6	Li et al. 2006
Effigia okeeffeae	Poposauroidea	late Norian-Rhaetian	209.5	Nesbitt 2011
Desmatosuchus smalli	Aetosauria	? late Norian	213.1	Small 2002
Stenomyti huangae	Aetosauria	late Norian-Rhaetian	209.5	Small and Martz 2013

Stagonolepis robertsoni	Aetosauria	late Carnian-earliest Norian	228.95	Sulej 2010
Stagonolepis olenkae	Aetosauria	late Carnian	229.5	Sulej 2010
Neoaetosauroides engaeus	Aetosauria	middle Norian	216	Desojo and Baez 2007
Aetosaurus ferratus	Aetosauria	late Norian	213.1	Schoch 2007
Revueltosaurus callenderi	Aetosauria	Norian-Rhaetian	213.65	Nesbitt 2011
Postosuchus kirkpatricki	Rauisuchidae	Norian-Rhaetian	213.65	Weinbaum 2011
Prestosuchus chiniquiensis	Loricata	Ladinian-earliest Carnian	237	Sues and Fraser 2010
Saurosuchus galilei	Loricata	late Carnian-earliest Norian	228.95	Nesbitt 2011
Batrachotomus kuperferzellensis	Loricata	late Ladinian (Longobardian)	238.25	Nesbitt 2011
Dromicosuchus grallator	Crocodylomorpha	early Norian	222.35	Nesbitt 2011
Protosuchus richardsoni	Crocodylomorpha	Hettangian	200.3	Nesbitt 2011
Dibothrosuchus elaphros	Crocodylomorpha	?Hettangian-Sinemurian	196.05	Wu and Chatterjee 1993
Sphenosuchus acutus	Crocodylomorpha	Hettangian-Sinemurian	196.05	Nesbitt 2011
Pelagosaurus typus	Crocodylomorpha	early Toarcian	179.3	Pierce and Benton 2006
Eudimorphodon ranzii	Pterosauria	late Norian	210	Nesbitt 2011
Campylognathoides spp.	Pterosauria	early Toarcian	182.5	Wellnhofer 1978 (modified)
Dorygnathus banthensis	Pterosauria	early Toarcian	182.5	Wellnhofer 1978 (modified)
Tawa hallae	Theropoda	late Norian-Rhaetian	209.5	Nesbitt 2011
Herrerasaurus ischigualastensis	Theropoda	late Carnian-earliest Norian	228.95	Nesbitt 2011
Coelophysis bauri	Theropoda	late Norian-Rhaetian	209.5	Nesbitt 2011
‘Syntarsus’ kayentakatae	Theropoda	Sinemurian-Pliensbachian	191	Tykoski 2005
Zupaysaurus rougieri	Theropoda	middle Norian	216	Ezcurra 2007
Dilophosaurus wetherilli	Theropoda	Sinemurian-Pliensbachian	191	Rauhut 2003

Eoraptor lunensis	Sauropodomorpha	late Carnian-earliest Norian	228.95	Sereno et al. 2013
Adeopapposaurus mognai	Sauropodomorpha	Early Jurassic	187.7	Martinez 2009
Plateosaurus spp.	Sauropodomorpha	late Norian-Rhaetian	209.65	Yates 2003
Melanorosaurus readi	Sauropodomorpha	middle Norian-Rhaetian	214.15	Yates 2007
Unaysaurus tolentinoi	Sauropodomorpha	early Norian	222.45	Leal et al. 2004
Pampadromaeus barberenai	Sauropodomorpha	late Carnian-earliest Norian	228.95	Cabreira et al. 2011
Jingshanosaurus xinwaensis	Sauropodomorpha	Hettangian	200.3	Yates 2012
Massospondylus carinatus	Sauropodomorpha	Hettangian-Sinemurian	196.05	Gow et al. 1990
Lesothosaurus diagnosticus	Ornithischia	Hettangian-Sinemurian	196.05	Nesbitt 2011
Heterodontosaurus tucki	Ornithischia	Hettangian-Sinemurian	196.05	Norman et al. 2011
Emausaurus ernsti	Ornithischia	Toarcian	182.5	Haubold 1990

Table S2. List of additional taxa from the Middle and Late Jurassic and Cretaceous used to calculate ancestral shapes falling into the Early Jurassic in the present analyses with data of occurrences (in million of years) and sources of skull reconstructions.

Species	Clade	Age	Time	Source
Dakosaurus andiniensis	Crocodylomorpha	Tithonian-Berriasian	145.95	Pol and Gasparini 2009
Isisfordia duncani	Crocodylomorpha	Albian-Cenomanian	99.4	Salisbury et al. 2006
Mahajangasuchus insignis	Crocodylomorpha	Maastrichtian	69.05	Turner and Buckley 2008
Sarcosuchus imperator	Crocodylomorpha	Berriasian-Cenomanian	119.25	Sereno et al. 2001
Montealtosuchus arrudacamposi	Crocodylomorpha	Turonian-Santonian	88.75	Carvalho et al. 2007
Leidyosuchus canadensis	Crocodylomorpha	Campanian	77.1	Wu et al. 2001
Hamadasuchus rebouli	Crocodylomorpha	Albian-Cenomanian	103.25	Larsson and Sues 2007
Kaprosuchus saharicus	Crocodylomorpha	Cenomnanian	97.2	Sereno and Larrson 2009
Notosuchus terrestris	Crocodylomorpha	Santonian	84.95	Fiorelli and Calvo 2008
Mariliasuchus amarali	Crocodylomorpha	Campanian-Maastrichtian	74.8	Zaher et al. 2006
Malawisuchus mwakasyungutiensis	Crocodylomorpha	Aptian	119	Gomani 1997
Gobiosuchus kielanae	Crocodylomorpha	Campanian	77.1	Osmólska et al. 1997
Araripesuchus wegeneri	Crocodylomorpha	Aptian-Albian	112.75	Sereno and Larsson 2009
Simosuchus clarki	Crocodylomorpha	Maastrichtian	69.05	Kley et al. 2010
Anatosuchus minor	Crocodylomorpha	Aptian-Albian	112.75	Sereno and Larsson 2009
Campinasuchus dinizi	Crocodylomorpha	Turonian-Santonian	88.75	Carvalho et al. 2011
Scaphognathus crassirostris	Pterosauria	Tithonian	148.55	Bennett 2014
Monolophosaurus jiangi	Theropoda	Callovian	164.8	Rauhut 2003
Haplocheirus sollers	Theropoda	Oxfordian	160.4	photo material by CF

Guanlong wucaii	Theropoda	Oxfordian	160.4	Xu et al. 2006a
Sinraptor dongi	Theropoda	Oxfordian	160.4	Currie and Zhao 1993
Allosaurus fragilis	Theropoda	Kimmeridgian-Tithonian	151.15	Loewen 2009
Ceratosaurus nasicornis	Theropoda	Kimmeridgian-Tithonian	151.15	Sampson and Witmer 2007
Limusaurus inextricabilis	Theropoda	Oxfordian	160.4	Xu et al. 2009
Compsognathus longipes	Theropoda	Tithonian	148.55	Peyer 2006
Archaeopteyx lithographica	Theropoda	Tithonian	148.55	Rauhut 2014
Shunosaurus lii	Sauropodomorpha	Bajocian-Callovian	166.9	Rauhut et al. 2011
Mamenchisaurus youngi	Sauropodomorpha	Oxfordian	160.4	Ouyang and Ye 2002
Diplodocus spp.	Sauropodomorpha	Kimmeridgian-Tithonian	151.15	Wilson and Sereno 1998
Camarasaurus lentus	Sauropodomorpha	Kimmeridgian-Tithonian	151.15	Wilson and Sereno 1998
Giraffatitan brancai	Sauropodomorpha	Kimmeridgian-Tithonian	151.15	Wilson and Sereno 1998
Huayangosaurus taibaii	Ornithischia	Bajocian-Callovian	166.9	Sereno and Dong 1992
Stegosaurus stenops	Ornithischia	Kimmeridgian-Tithonian	151.15	Sereno and Dong 1992
Dysalotosaurus lettowvorbecki	Ornithischia	upper Kimmeridgian	153.25	Hübner and Rauhut 2010
Agilisaurus louderbacki	Ornithischia	Bajocian-Callovian	166.9	Barrett et al. 2005
Yinlong downsi	Ornithischia	Oxfordian	160.4	Xu et al. 2006b
Camptosaurus dispar	Ornithischia	Kimmeridgian-Tithonian	151.15	Brill and Carpenter 2006
Psittacosaurus mongoliensis	Ornithischia	Lower Cretaceous	122.75	Sereno et al. 1988

Table S3. Additional taxa for time calibration with data of occurrences (in millions of years).

Species	Clade	Age	Time
Noteosuchus colletti	Rhynchosauria	Induan-early Olenekian	250.7
Archosaurus rossicus	Proterosuchidae	Changhsingian	253.15
Dorosuchus neoetus	Archosuriformes	Anisian	244.6
Aetosauroides scagliai	Aetosauria	late Carnian-earliest Norian	228.95
Turfanosuchus dabanensis	Gracilisuchidae	late Anisian	243.3
Ticinosuchus ferox	Suchia	late Anisian	243.3
Ctenosauriscus koeneni	Poposauroidea	late Olenekian	248.2
Orthosuchus stormbergi	Crocodyliformes	Hettangian-Sinemurian	196.05
Hsisosuchus dashanpuensis	Crocodyliformes	Bajocian-Callovian	166.9
Calsoyasuchus valliceps	Crocodyliformes	Sinemurian-Pliensbachian	191
Asilisaurus kongwe	Ornithodira	late Anisian	243.3
Pisanosaurus mertii	Ornithischia	late Carnian-earliest Norian	228.95
Eocursor parvus	Ornithischia	middle Norian-Rhaetian	214.15
Scelidosaurus harrisonii	Ornithischia	Sinemurian	195.05
Vulcanodon karibaensis	Sauropodomorpha	Hettangian	200.3
Camposaurus arizonensis	Theropoda	early Norian	221.15
Berberosaurus liassicus	Theropoda	Pliensbachian-Toarcian	182.45
Proceratosaurus bradleyi	Theropoda	Bathonian	166.2

2. Description of landmarks and semi-landmarks

Table S4. Description of landmarks and semi-landmark description. Semi-landmarks (semi-LMs) are marked with an 'S' (Fig. S1).

LMs	Descrciption
1	Most anterior point of the premaxilla along the tooth row.
2	Contact of the lateral wall of premaxilla and maxilla along the tooth row (thereby making it possible to capture the subnarial notches of *Heterodontosaurus*, *Protrosuchus* and *Tawa*).
3	Contact between maxilla and jugal along the ventral margin of the skull.
4	Most posterior point of the lateral condyle of the quadrate (if the quadrate is covered by the quadratojugal, the most posterior point of the posteroventral corner of the quadratojugal is chosen).
5	The most posterodorsal point of the subnarial process of the premaxilla and the maxilla. (The position of this landmark in the reconstruction of *Yinlong* based on personal observations).
6	The most posterior point of the ascending process of the maxilla along the dorsal margin of the antorbital fenestra. If no antorbital fenestra is developed, the most anterodorsal contact between maxilla and lacrimal was marked.
7	The most posterior point of the jugal process of the maxilla along the ventral margin of the antorbital fenestra. If no antorbital fenestra is developed, the most posteroventral contact between the maxilla and lacrimal was marked (The position of this landmark in the reconstruction of *Allosaurus* based on personal observations of various specimens of *Allosaurus*).
8	The contact between jugal and lacrimal along the orbital margin (for *Azendohsaurus*, *Malawisuchus*, *Mariliasuchus*, *Protosuchus* and *Trilophosaurus*, the most anterior point of the jugal along the orbital margin was chosen as the lacrimal is excluded from the orbit. In contrast, for some phytosaurs the most ventral point of the lacrimal along the orbital margin was marked, as the jugal is excluded from the orbit. The position of the landmark in the reconstruction of *Scaphognathus* is based on Wellnhofer 1975).
9	Contact between jugal and postorbital along the orbital margin.
10	The most dorsal point of the postorbital along the orbital margin.
11	The contact between jugal and postorbital along the margin of the lateral temporal fenestra/opening. For *Simosuchus* we plotted the most dorsal contact of the jugal along the anterodorsal margin of the lateral temporal fenestra.
12	The ventral contact between postorbital and squamosal (This contact is usually located along the dorsal margin of the lateral temporal fenestra/opening, but is different in some rhynchosaurs, aetosaurs, *Dakosaurus*, *Postosuchus, Protosuchus* and *Yinlong*).
13	The dorsal contact between postorbital and squamosal.
14	Most ventral contact between the posterior process of the squamosal and quadrate.

15	Contact between jugal and quadratojugal along the ventral margin of the lateral temporal fenestra (For those taxa where the fenestra is ventrally open due to a reduction of the quadratojugal, the most posterior point of the posterior process of the jugal was marked. In the reconstruction of *Dysalotosaurus*, we plotted this landmark at the most posterior point of the jugal along the ventral margin of the lateral temporal fenestra).
S16-S17	Two semi-landmarks between LM1 and LM2 along the ventral margin of the premaxilla.
S18-S20	Three semi-landmarks between LM2 and LM3 along the ventral margin of the maxilla.
S21-S35	15 semi-landmarks between LM1 and LM14 along the dorsal margin of the skull
S36-S38	Four semi-landmarks between LM6 and LM7 along the anterior and posterior margin of the antorbital fenestra. In taxa lacking am antorbital fenestra, the semi-landmarks are placed along the suture contact of maxilla and lacrimal (Fig. S1).
S39-S43	Four semi-landmarks between LM8 and LM10 along the anterodorsal margin of the orbit.
S44-S45	Two semi-landmarks between LM10 and LM9 along the posterior margin of the orbit.
S46	One semi-landmark between LM8 and LM9 along the ventral margin of the orbit.
S47-S48	Two semi-landmarks between LM11 and LM15 along the anteroventral margin of the lateral temporal opening.
S49-S53	Five semi-landmarks between LM12 and LM14 along the ventral margin of the squamosal.

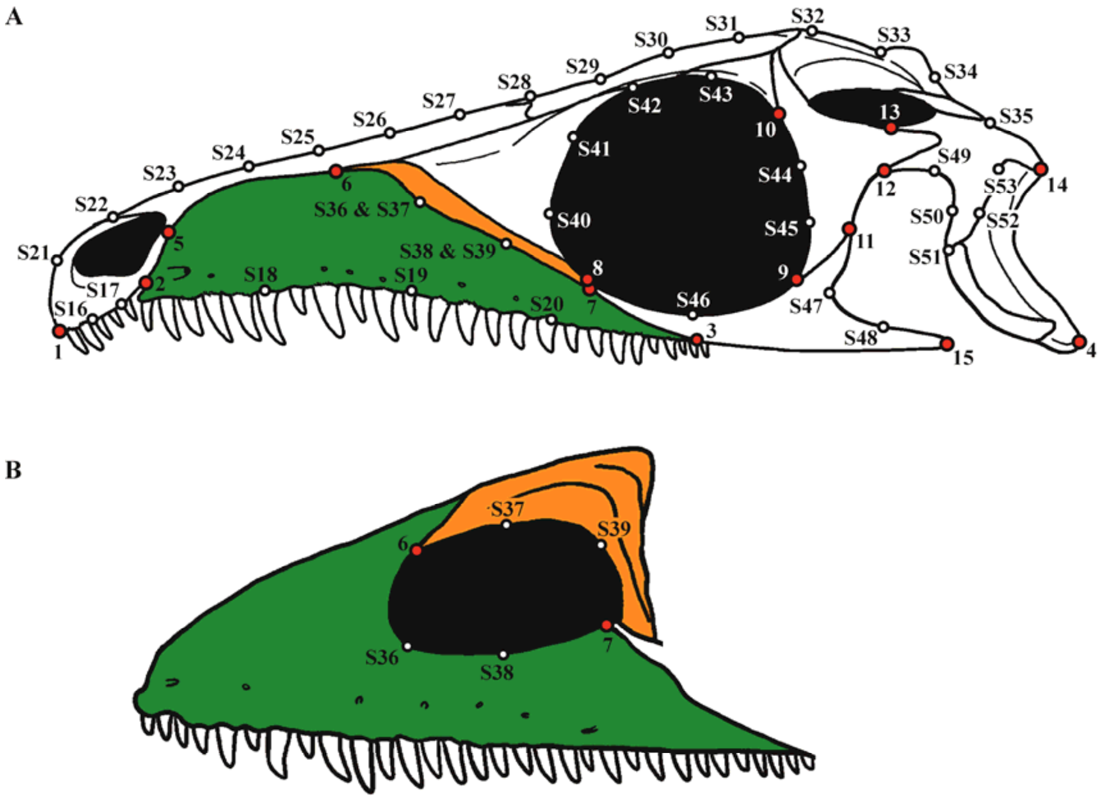

Figure S1. Distribution of landmarks and semi-landmarks. **(A)** Skull reconstruction of *Prolacerta broomi* in lateral view. Due to the absence of an antorbital fenestra the semi-landmarks S36 to S39 are placed along the suture contact of maxilla (green) and lacrimal (orange). **(B)** Reconstruction of maxilla (green) and lacrimal (orange) in anatomical position of *Proterosuchus fergusi* in lateral view showing the position of semi-landmarks S36 to S39 when an antorbital fenestra is present. All reconstructions are modified after Ezcurra and Butler (2015).

3. Phylogeny

To reconstruct ancestral shapes, we created two informal, time-calibrated supertrees based on recent literature. The main topology was based on Nesbitt (2011), but for one tree the position of phytosaurs were placed within crown Archosauria (Sereno 1991; Brusatte et al. 2010; Ezcurra 2016), while in the other phytosaurs are treated as non-archosaurian archosauromorphs following Nesbitt (2011). The sources of the phylogenetic positions of taxa not included in the analyses of Nesbitt (2011) are listed in Table S5.

Table S5. Sources for phylogenetic position of taxa included in the informal supertree.

Species	Clade	Source of Phylogeny
Langobardisaurus pandolfii	‘Protorosauria’	Pritchard et al. 2015
Protorosaurus speneri	‘Protorosauria’	Gottman-Quesada and Sander 2009
Tanystropheus longobardicus	‘Protorosauria’	Ezcurra et al. 2014
Prolacerta broomi	Prolacertidae	Ezcurra et al. 2014
Proterosuchus fergusi	Proterosuchidae	Ezcurra et al. 2014
Proterosuchus alexanderi	Proterosuchidae	Ezcurra et al. 2014
Proterosuchus goweri	Proterosuchidae	Ezcurra et al. 2014
Trilophosaurus buettneri	Allokotosauria	Ezcurra et al. 2014
Azendohsaurus madagaskarensis	Allokotosauria	Nesbitt et al. 2013
Bentonyx sidensis	Rhynchosauria	Langer et al. 2010
Hyperodapedon gordoni	Rhynchosauria	Langer and Schultz 2000
Hyperodapedon huxleyi	Rhynchosauria	Hone and Benton 2008
Hyperodapedon sanjuanensis	Rhynchosauria	Hone and Benton 2008
Mesosuchus browni	Rhynchosauria	Hone and Benton 2008
Rhynchosaurus articeps	Rhynchosauria	Hone and Benton 2008
Rhynchosaurus brodiei	Rhynchosauria	Hone and Benton 2008
Teyumbaita sulcognathus	Rhynchosauria	Hone and Benton 2008
Garjainia prima	Erythrosuchidae	Pers. obs.
Shansisuchus shansisuchus	Erythrosuchidae	Gower and Sennikov 1996
Gualosuchus reigi	Proterochampsidae	Dilkes and Arcucci 2012
Smilosuchus gregorii	Phytosauria	Stocker 2010

Brachysuchus megalodon	Phytosauria	Stocker 2010
Leptosuchus adamanensis	Phytosauria	Stocker 2010
Leptosuchus studeri	Phytosauria	Stocker 2010
Mystriosuchus planirostris	Phytosauria	Parker and Irmis 2006
Mystriosuchus westphali	Phytosauria	Parker and Irmis 2006
Nicrosaurus kapffi	Phytosauria	Parker and Irmis 2006
Pseudopalatus pristinus	Phytosauria	Parker and Irmis 2006
Gracilisuchus stipanicicorum	Suchia	Butler et al. 2014
Desmatosuchus smalli	Aetosauria	Desojo et al. 2012
Stagonolepis robertsoni	Aetosauria	Desojo et al. 2012
Stagonolepis olenkae	Aetosauria	Desojo et al. 2012
Neoaetosauroides engaeus	Aetosauria	Desojo et al. 2012
Aetosaurus ferratus	Aetosauria	Desojo et al. 2012
Revueltosaurus callenderi	Aetosauria	Desojo et al. 2012
Stenomyti huangae	Aetosauria	Small and Martz 2013
Dakosaurus andiniensis	Crocodylomorpha	Bronzati et al. 2012
Isisfordia duncani	Crocodylomorpha	Bronzati et al. 2012
Leidyosuchus canadensis	Crocodylomorpha	Bronzati et al. 2012
Pelagosaurus typus	Crocodylomorpha	Bronzati et al. 2012
Sarcosuchus imperator	Crocodylomorpha	Bronzati et al. 2012
Araripesuchus wegeneri	Crocodylomorpha	Pol et al. 2014; Sertich and O'Connor 2014
Campinasuchus dinizi	Crocodylomorpha	Pol et al. 2014
Gobiosuchus kielanae	Crocodylomorpha	Pol et al. 2014; Sertich and O'Connor 2014
Hamadasuchus rebouli	Crocodylomorpha	Pol et al. 2014; Sertich and O'Connor 2014
Kaprosuchus saharicus	Crocodylomorpha	Pol et al. 2014; Sertich and O'Connor 2014
Mahajangasuchus insignis	Crocodylomorpha	Pol et al. 2014; Sertich and O'Connor 2014
Malawisuchus mwakasyungutiensis	Crocodylomorpha	Pol et al. 2014; Sertich and O'Connor 2014
Mariliasuchus amarali	Crocodylomorpha	Pol et al. 2014; Sertich and O'Connor 2014
Montealtosuchus arrudacamposi	Crocodylomorpha	Pol et al. 2014; Sertich and O'Connor 2014
Notosuchus terrestris	Crocodylomorpha	Pol et al. 2014; Sertich and O'Connor 2014
Simosuchus clarki	Crocodylomorpha	Pol et al. 2014; Sertich and O'Connor 2014
Anatosuchus minor	Crocodylomorpha	Sertich and O'Connor 2014
Eudimorphodon ranzii	Pterosauria	Andres and Myers 2013
Campylognathoides spp.	Pterosauria	Andres and Myers 2013
Dorygnathus banthensis	Pterosauria	Andres and Myers 2013
Scaphognathus crassirostris	Pterosauria	Andres and Myers 2013
'Syntarsus' kayentakatae	Theropoda	Sues et al. 2011
Zupaysaurus rougieri	Theropoda	Sues et al. 2011
Monolophosaurus jiangi	Theropoda	Carrano et al. 2012

Allosaurus fragilis	Theropoda	Carrano et al. 2012
Sinraptor dongi	Theropoda	Carrano et al. 2012
Archaeopteyx lithographica	Theropoda	Foth et al. 2014
Compsognathus longipes	Theropoda	Foth et al. 2014
Guanlong wucaii	Theropoda	Foth et al. 2014
Haplocheirus sollers	Theropoda	Foth et al. 2014
Ceratosaurus nasicornis	Theropoda	Pol and Rauhut 2012
Limusaurus inextricabilis	Theropoda	Pol and Rauhut 2012
Adeopapposaurus mognai	Sauropodomorpha	Martínez 2009
Eoraptor lunensis	Sauropodomorpha	Martínez et al. 2013
Pampadromaeus barberenai	Sauropodomorpha	Martínez et al. 2013
Jingshanosaurus xinwaensis	Sauropodomorpha	Yates 2007
Massospondylus carinatus	Sauropodomorpha	Yates 2007
Melanorosaurus readi	Sauropodomorpha	Yates 2007
Plateosaurus spp.	Sauropodomorpha	Yates 2007
Unaysaurus tolentinoi	Sauropodomorpha	Yates 2007
Shunosaurus lii	Sauropodomorpha	Wilson 2002
Mamenchisaurus youngi	Sauropodomorpha	Wilson 2002
Diplodocus spp.	Sauropodomorpha	Wilson 2002
Camarasaurus lentus	Sauropodomorpha	Wilson 2002
Giraffatitan brancai	Sauropodomorpha	Wilson 2002
Lesothosaurus diagnosticus	Ornithischia	Butler et al. 2010
Heterodontosaurus tucki	Ornithischia	Butler et al. 2010
Emausaurus ernsti	Ornithischia	Butler et al. 2010
Huayangosaurus taibaii	Ornithischia	Maidment et al. 2008
Stegosaurus stenops	Ornithischia	Maidment et al. 2008
Dysalotosaurus lettowvorbecki	Ornithischia	McDonald et al. 2010
Agilisaurus louderbacki	Ornithischia	Butler et al. 2010
Yinlong downsi	Ornithischia	Butler et al. 2010
Camptosaurus dispar	Ornithischia	Butler et al. 2010
Psittacosaurus mongoliensis	Ornithischia	Butler et al. 2010

The correlation between shape and phylogeny was tested using multivariate K statistics on the basis of Procrustes-fitted landmark coordinates using the program R (R Development Core Team) and the package geomorph (Adams and Otárola-Castillo 2013). This method estimates the strength of a phylogenetic signal in a data set in relation to a simulated Brownian motion model and was performed with 1,000 replications (Blomberg et al., 2003; Paradis, 2012; Adams, 2014). The strength of the phylogenetic signal is expressed as K and p-values. The test reveals that skull shape of archosauromorphs correlates significantly with phylogeny, while the position of phytosaurs has no impact on the signal (Table S6). However, the K-value < 1 implies that the skull shapes of the terminal taxa resemble each other less than expected under Brownian motion evolution (Blomberg et al., 2003). To confirm these results we performed an additional permutation test in MorphoJ (Klingenberg 2011), shuffling the Procrustes-fitted landmark coordinates of each taxon randomly across the tree (10,000 times), while the topology is held constant (Laurin 2004; Klingenberg and Gidaszewski 2010). As found using K statistics, cranial shape correlates significantly with phylogeny as the squared length of the original supertree (= tree length) occurs in over 95% of the randomly generated trees (Fig. S3; Table S6).

Table S6. Strength of the phylogenetic signal of the skull shape of Archosauromorpha.

	K	p-value	Tree length	p-value
**Phytosauria as crown**	0.079	0.001	3.431	<0.001
**Phytosauria as stem**	0.079	0.001	3.429	<0.001

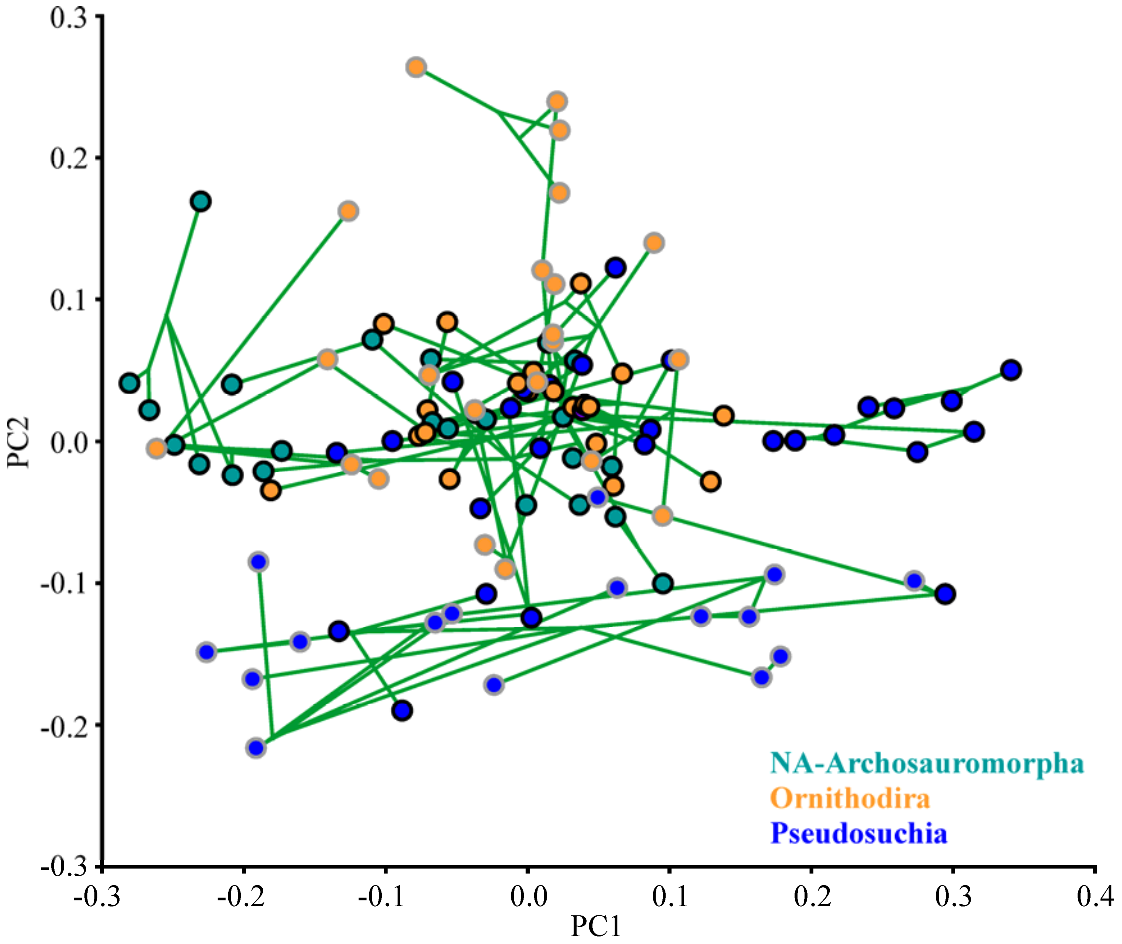

Figure S2. Morphospace of archosauromorphs, including post-Early Jurassic pseudosuchians (blue circle with grey outline) and ornithodirans (orange circle with grey outline), which were used specifically to trace ancestral shapes of archosaurs for the Early Jurassic period. Archosauromorphs living from the Permian to the end of Early Jurassic are marked with black outlines.

5. Disparity and NPMANOVA results

Table S7. Sum of variances of Archosauromorpha through time. The values for ornithodirans and dinosaurs are not affected by the phylogenetic position of phytosaurs. NA = non-archosaurian. Numbers in brackets show the sample size for each group per bin.

	all Archosauromorpha	Ornithodira	Dinosauria
PERMIAN (PE)	0.0023 (7)	-	-
EARLY TRIASSIC (ET)	0.0041 (20)	-	-
ANISIAN (ANI)	0.0217 (14)	-	-
LADINIAN (LAD)	0.0147 (11)	-	-
CARNIAN (CAR)	0.0415 (27)	0.0031 (9)	0.0031 (9)
NORIAN1 (NOR1)	0.0365 (17)	0.0035 (6)	0.0035 (6)
NORIAN2 (NOR2)	0.0362 (30)	0.0081 (11)	0.0068 (9)
HETTANGIAN (HET)	0.0118 (5)	-	-
SINEMURIAN (SIN)	0.0267 (14)	0.0222 (9)	0.0222 (9)
TOARCIAN (TOA)	0.0326 (8)	0.0121 (5)	-
**Phytosaurs as crown group**	**NA-Archosauromorpha**	**Archosauria**	**Pseudosuchia**
PERMIAN (PE)	0.0023 (7)	-	-
EARLY TRIASSIC (ET)	0.0053 (13)	0.0000 (7)	0.0000 (6)
ANISIAN (ANI)	0.0188 (12)	-	-
LADINIAN (LAD)	0.0207 (5)	0.0068 (6)	0.0068 (6)
CARNIAN (CAR)	0.0064 (6)	0.0176 (21)	0.0180 (12)
NORIAN1 (NOR1)	-	0.0290 (16)	0.0287 (10)
NORIAN2 (NOR2)	0.0282 (NOR1+NOR2) (3)	0.0345 (28)	0.0431 (17)
HETTANGIAN (HET)	-	0.0118 (5)	0.0001 (4)
SINEMURIAN (SIN)	-	0.0267 (14)	0.0089 (5)
TOARCIAN (TOA)	-	0.0326 (8)	0.0000 (3)
**Phytosaurs as stem group**	**NA-Archosauromorpha**	**Archosauria**	**Pseudosuchia**
PERMIAN (PE)	0.0023 (7)	-	-
EARLY TRIASSIC (ET)	0.0051 (14)	0.0000 (6)	0.0000 (5)
ANISIAN (ANI)	0.0188 (12)	-	-
LADINIAN (LAD)	0.0207 (5)	0.0068 (6)	0.0068 (6)
CARNIAN (CAR)	0.0815 (8)	0.0076 (19)	0.0044 (10)
NORIAN1 (NOR1)	0.0317 (8)	0.0106 (9)	0.0000 (3)
NORIAN2 (NOR2)	0.0398 (10)	0.0153 (20)	0.0171 (9)
HETTANGIAN (HET)	-	0.0118 (5)	0.0001 (4)
SINEMURIAN (SIN)	-	0.0267 (14)	0.0089 (5)
TOARCIAN (TOA)	-	0.0326 (8)	0.0000 (3)

Table S8. Statistical differences of sum of variances and morphospace position between subsequent time bins for all Archosauromorpha. Bold p-values represent significant results.

	Sum of Variance		NPMANOVA	
	**Difference**	**p-value**	**F**	**p-value**
**PE-ET**	0.0018	0.4041	6.9180	**0.0495**
**ET-ANI**	0.0176	**0.0002**	16.9400	**0.0045**
**ANI-LAD**	-0.0070	0.0717	3.85900	0.9180
**LAD-CAR**	0.0268	0.0870	0.9132	1.0000
**CAR-NOR1**	-0.0050	0.7068	4.6600	0.4860
**NOR1-NOR2**	-0.0004	0.9720	0.9306	1.0000
**NOR2-HET**	-0.0243	0.1264	8.2920	0.0765
**HET-SIN**	0.0149	**0.0342**	3.4680	1.0000
**SIN-TOA**	0.0059	0.6516	6.6270	**0.0360**

Table S9. Statistical differences of sum of variances and morphospace position between subsequent time bins for non-archosaurian Archosauromorpha. Bold p-values represent significant results.

	Sum of Variance		NPMANOVA	
**Phytosauria as crown**	**Difference**	**p-value**	**F**	**p-value**
**PE-ET**	0.0030	0.2364	7.0960	**0.0390**
**ET-ANI**	0.0135	**0.0011**	17.3400	**0.0015**
**ANI-LAD**	0.0019	0.7939	3.7280	0.4110
**LAD-CAR**	-0.0143	0.3878	23.2200	**0.0435**
**CAR-NOR (NOR1+NOR2)**	0.0218	0.1881	4.8530	0.1830
**Phytosauria as stem**				
**PE-ET**	0.0028	0.2675	7.1040	**0.0294**
**ET-ANI**	0.0138	**0.0015**	19.6300	**0.0021**
**ANI-LAD**	0.0019	0.7950	3.7280	0.5712
**LAD-CAR**	0.0607	**0.0287**	2.1510	1.0000
**CAR-NOR1**	-0.0497	**0.0001**	7.0120	0.7371
**NOR1-NOR2**	0.0081	0.7470	0.4481	1.0000

Table S10. Sum of variances of non-archosaurifom (NAF-) Archosauromorpha and non-archosaurian (NA-) Archosauriformes through time.

Phytosaurs as crown group	NAF-Archosauromorpha	NA-Archosauriformes
PERMIAN (PE)	0.0025	-
EARLY TRIASSIC (ET)	-	0.0026
ANISIAN (ANI)	0.0125	0.0126
LADINIAN (LAD)	-	0.0013
CARNIAN (CAR)	0.0064	-
**Phytosaurs as stem group**	**NAF-Archosauromorpha**	**NA-Archosauriformes**
PERMIAN (PE)	0.0025	-
EARLY TRIASSIC (ET)	-	0.0026
ANISIAN (ANI)	0.0125	0.0126
LADINIAN (LAD)	-	0.0012
CARNIAN (CAR)	0.0064	-
NORIAN1 (NOR1)	-	0.0022
NORIAN2 (NOR2)	-	0.0062

Table S11. Statistical differences of sum of variances and morphospace position between subsequent time bins for non-archosaurifom (NAF-) Archosauromorpha and non-archosaurian (NA-) Archosauriformes when phytosaurs are members of the crown-group of Archosauria. Bold p-values represent significant results.

	Sum of Variance		NPMANOVA	
**NAF-Archosauromorpha**	**Difference**	**p-value**	**F**	**p-value**
PE- ANI	0.0100	**0.0240**	17.9200	**0.0048**
ANI-CAR	-0.0061	0.5579	16.9000	**0.0069**
**NA-Archosauriformes**				
ET-ANI	0.0100	**0.0234**	4.6460	**0.0054**
ANI-LAD	-0.0113	0.1972	7.1130	0.3048

Table S12. Statistical differences of sum of variances and morphospace position between subsequent time bins for non-archosaurifom (NAF-) Archosauromorpha and non-archosaurian (NA-) Archosauriformes when phytosaurs are members of the stem-line of Archosauria. Bold p-values represent significant results.

	Sum of Variance		NPMANOVA	
**NAF-Archosauromorpha**	**Difference**	**p-value**	**F**	**p-value**
**PE- ANI**	0.0100	**0.0271**	17.9200	**0.0048**
**ANI-CAR**	-0.0061	0.5574	16.9000	**0.0069**
**NA-Archosauriformes**				
**ET-ANI**	0.0100	**0.0148**	4.5880	**0.0350**
**ANI-LAD**	-0.0113	0.1995	7.1130	1.0000
**LAD -NOR1**	0.0010	0.2452	59.4100	**0.0920**
**NOR1-NOR2**	0.0039	0.1104	1.8820	1.0000

Table S13. Statistical differences of sum of variances and morphospace position between subsequent time bins for Archosauria. Bold p-values represent significant results.

	Sum of Variance		NPMANOVA	
**Phytosauria as crown**	**Difference**	**p-value**	**F**	**p-value**
**ET-LAD**	0.0067	**0.0003**	8.2230	0.1372
**LAD-CAR**	0.0108	0.5922	3.1740	1.0000
**CAR-NOR1**	0.0113	0.1035	4.8790	0.4620
**NOR1-NOR2**	0.0055	0.3742	0.8775	1.0000
**NOR2-HET**	-0.0226	0.1507	9.4540	**0.0196**
**HET-SIN**	0.0149	**0.0367**	3.4680	0.7643
**SIN-TOA**	0.0059	0.6574	6.6270	**0.0280**
**Phytosauria as stem**				
**ET-LAD**	0.0067	**0.0013**	6.8150	0.4592
**LAD-CAR**	0.0008	0.6504	4.5100	0.1596
**CAR-NOR1**	0.0030	0.1618	4.7660	0.0728
**NOR1-NOR2**	0.0047	0.2585	2.2570	1.0000
**NOR2-HET**	-0.0035	0.7053	14.0200	**0.0056**
**HET-SIN**	0.0149	**0.0355**	3.4680	0.7027
**SIN-TOA**	0.0059	0.6568	6.6270	**0.0168**

Table S14. Statistical differences of sum of variances and morphospace position between subsequent time bins for Pseudosuchia. Bold p-values represent significant results.

	Sum of Variance		NPMANOVA	
**Phytosauria as crown**	**Difference**	**p-value**	**F**	**p-value**
**ET-LAD**	0.0068	**0.0019**	6.9330	0.4368
**LAD-CAR**	0.0112	0.6098	6.1220	0.1176
**CAR-NOR1**	0.0107	0.0921	4.5650	0.8427
**NOR1-NOR2**	0.0144	0.1099	1.2660	1.0000
**NOR2-HET**	-0.0430	**0.0079**	9.0340	**0.0168**
**HET-SIN**	0.0088	**0.0180**	9.3810	0.4956
**SIN-TOA**	-0.0089	0.6786	35.4500	0.4424
**Phytosauria as stem**				
**ET-LAD**	0.0068	**0.001900**	5.5630	0.4564
**LAD-CAR**	-0.0024	0.441700	9.2180	**0.0224**
**CAR-NOR1**	-0.0044	0.618500	22.4700	0.1148
**NOR1-NOR2**	0.0171	0.102700	5.2420	0.1596
**NOR2-HET**	-0.0170	0.069800	14.7400	**0.0336**
**HET-SIN**	0.0088	**0.016000**	9.3810	0.4760
**SIN-TOA**	-0.0089	0.900600	35.4500	0.5375

Table S15. Statistical differences of sum of variances and morphospace position between subsequent time bins for Ornithodira/Dinosauria. Bold p-values represent significant results.

	Sum of Variance		NPMANOVA	
**Ornithodira**	**Difference**	**p-value**	**F**	**p-value**
**CAR-NOR1**	0.0004	0.8045	8.1730	**0.0220**
**NOR1-NOR2**	0.0046	0.0822	0.5507	1.0000
**NOR2-SIN**	0.0140	**0.0299**	3.7450	0.1870
**SIN-TOA**	-0.0100	0.2724	3.2080	0.2290
**Dinosauria**				
CAR-NOR1	0.0004	0.7908	8.1730	**0.0114**
NOR1-NOR2	0.0033	0.2235	0.7489	1.0000
NOR2-SIN	0.0154	**0.0351**	3.6580	0.1950

Table S16. Statistical differences of sum of variances and morphospace position between different groups within time bin when phytosaurs are crown-archosaurs. Bold p-values represent significant results. NAF = non-archosauriform; NA = non-archosaurian.

**Phytosauria as crown**	**Sum of Variance**		**NPMANOVA**	
**NA-Archosauromorpha vs. Archosauria**	**Difference**	**p-value**	**F**	**p-value**
**EARLY TRIASSIC**	0.0053	**0.0315**	3.8020	**0.0354**
**LADINIAN**	0.0140	**0.0200**	2.3470	0.3189
**CARNIAN**	-0.0112	0.6621	45.2500	**0.0010**
**NAF-Archosauromorpha vs. NA-Archosauriformes**				
**ANISIAN**	-0.0001	0.9834	6.5960	**0.0051**
**NA-Archosauromorpha vs. Pseudosuchia**				
**EARLY TRIASSIC**	0.0053	**0.0355**	3.3630	0.0642
**LADINIAN**	0.0140	**0.0149**	2.3470	0.3231
**CARNIAN**	-0.0116	0.6686	50.1200	**0.0010**
**NA-Archosauromorpha vs. Ornithodira**				
**CARNIAN**	0.0033	0.7244	100.4000	**0.0020**
**Pseudosuchia vs. Ornithodira**				
**CARNIAN**	0.0149	0.4270	11.0200	**0.0010**
**NORIAN1**	0.0252	**0.0002**	8.0490	**0.0168**
**NORIAN2**	0.0349	**< 0.0001**	5.4060	**0.0330**
**SINEMURIAN**	-0.0133	0.1795	7.5870	**0.0012**
**TOARCIAN**	-0.0121	0.4856	22.2400	**0.0190**

Table S17. Statistical differences of sum of variances and morphospace position between different groups within time bin when phytosaurs are stem-archosaurs. Bold p-values represent significant results. NAF = non-archosauriform; NA = non-archosaurian.

**Phytosaurs as stem**	**Sum of Variance**		**NPMANOVA**	
**NA-Archosauromorpha vs. Archosauria**	**Difference**	**p-value**	**F**	**p-value**
**EARLY TRIASSIC**	0.0050	**0.0433**	3.0990	0.0849
**LADINIAN**	0.0140	**0.0177**	2.3470	0.3186
**CARNIAN**	0.0739	**< 0.0001**	13.1600	**0.0020**
**NORIAN1**	0.0211	0.1338	13.5700	**0.0060**
**NORIAN2**	0.0245	0.0058	17.1800	**0.0010**
**NAF-Archosauromorpha vs. NA-Archosauriformes**				
**ANISIAN**	0.0001	0.9870	6.5960	**0.0044**
**NA-Archosauromorpha vs. Pseudosuchia**				
**EARLY TRIASSIC**	0.0050	0.0555	2.7050	0.1503
**LADINIAN**	0.0140	**0.0144**	2.3470	0.3246
**CARNIAN**	0.0771	**< 0.0001**	8.9560	**0.0420**
**NORIAN1**	0.0317	0.4941	8.4390	0.2670
**NORIAN2**	0.0228	**0.0286**	11.7100	**0.0180**
**NA-Archosauromorpha vs. Ornithodira**				
**CARNIAN**	0.0784	**0.0002**	6.6580	0.0610
**NORIAN1**	0.0282	**0.0452**	10.6200	**0.0460**
**NORIAN2**	0.0317	**0.0005**	12.7700	**0.0110**
**Pseudosuchia vs. Ornithodira**				
**CARNIAN**	0.0013	0.3593	19.2600	**0.0010**
**NORIAN1**	-0.0035	0.4831	26.9500	0.1280
**NORIAN2**	0.0089	0.0502	6.0580	**0.0010**
**SINEMURIAN**	-0.0133	0.1766	7.5870	**0.0009**
**TOARCIAN**	-0.0121	0.4835	22.2400	**0.0149**

5. Additional results and discussion points

5.1. General disparity trend within archosauromorph cranium

The current study shows that after the end-Permian mass extinction, cranial disparity of archosauromorphs increased slowly during the Early Triassic, which is in agreement with a general delayed recovery of ecosystems and taxon diversity after the extinction event (Irmis and Whiteside 2012; Lau et al. 2016). However, the shift in morphospace (Table S8) already indicates an initial diversification of archosauromorphs starting in the Early Triassic. The Anisian peak, which corresponds to a shift in morphospace, is evidence of an abrupt radiation of archosauromorphs (including crown archosaurs), beginning in the Middle Triassic and continuing until the end of the Late Triassic (Nesbitt 2011, Sidor et al. 2013) leading to a diversification of cranial morphologies including taxa with long (e.g. Phytosauria, Coelophysidae, Thalattosuchia, Protochampsidae) and short (e.g. Rhynchosauria, Heterodontosauridae, *Lotosaurus*) snouts. When compared to each other, non-archosauriform archosauromorphs are similar in cranial disparity to non-archosaurian archosauriforms during the Anisian, but occupy different regions in morphospace (Table S16, S17), supporting a process of radiation during the Middle Triassic. This radiation corresponded also with an increase in body size (Sookias et al. 2012a,b) and reveals an ecological diversification into different niches and probable dietary preferences, including piscivory (e.g. Phytosauria, Proterochampsia), omnivory (basal Sauropodomorpha, Aetosauria), carnivory (e.g. Rauisuchidae, Theropoda) and herbivory (e.g. Rhynchosauria, Ornithischia) (see Butler et al. 2011; Nesbitt et al. 2013). The mass extinction at the end of the Triassic resulted in a decrease in taxonomic diversity, including the extinction of all stem line archosaurs and all non-crocodylomorph pseudosuchians, which in turn led to a decrease in cranial disparity.

During their coexistence, the cranial morphospace of non-archosaurian archosauromorphs and archosaurs overlaps during the Ladinian (this result is found with both phylogenetic positions of phytosaurs, Table S16, S17). However, this overlap is not unambiguous evidence for ecological competition between the two groups during this period, but could also result from a statistical artefact as the crown group members in this time bin tend not to show extremes of skull shape (e.g. Gracilisuchidae and various basal Loricata), while the stem line representatives include taxa with short and high (Allokotosauria) and long and flattened skulls (Protochampsia), resulting in similar medians (centroids) in morphospace. However, whether this overlap is a real signal needs to be tested in the future with more detailed analyses including multiple lines of evidence.

5.2. The effect of the phylogenetic position of Phytosauria

The disparity trajectories for non-archosaurian archosaurormorphs are greatly affected by the phylogenetic position of Phytosauria. When phytosaurs are considered the sister-taxon of Archosauria (Nesbitt 2011), the cranial disparity of non-archosaurian archosauromorphs increases from the late Permian to the Carnian, in which the changes between the Ladinian and the Carnian are significant. After a Carnian peak their cranial disparity decreases significantly in the early Norian, but re-expands slightly in the late Norian, before the group goes extinct at the end of the Late Triassic (Table S9). In contrast, the cranial disparity of Archosauria increases from the Early Triassic until the late Norian. From the late Norian into the Hettangian cranial disparity decreases, and then it expands again until the Toarcian (Fig. S3, Table S13). The changes between the Hettangian and Sinemurian are significant. In this scenario, cranial disparity of non-archosaurian archosauromorphs is higher than that of

Archosauria throughout the interval over which they coexist, but only significantly higher from the Early Triassic to the Carnian (Table S17). Non-archosaurian archosauromorphs exhibit a significant shift in morphospace from the late Permian to the Early Triassic and from the Early Triassic to the Anisian (Table S9), while in Archosauria these shifts occur from the late Norian to the Hettangian and from the Sinemurian to the Toarcian (Table S13). When compared to each other, both groups are significantly separated from each other in morphospace over the entire Late Triassic (Fig. S4), but not in the Early and Middle Triassic (Table S17). In sum, if phytosaurs were stem-archosaurs, non-archosaurian archosauromorph disparity far exceeds that of Archosauria during the whole Triassic, before the sudden extinction of all non-crown taxa at the end of the Triassic. In addition, the cranial disparity of Pseudosuchia and Ornithodira would be relatively equal during the Late Triassic (Fig. S3), although both groups occupy different areas of morphospace (as found by Brusatte et al. 2008). In this scenario, the “true” radiation of crown archosaurs began following the end-Triassic extinction, precipitated by an increase of taxonomic and morphological diversity in Ornithodira. Thus, our view of the evolutionary radiation of Archosauria, pseudosuchians and ornithodirans in particular, is strongly affected by the inclusion or exclusion of phytosaurs within the crown. This in turn highlights the importance of phylogeny in understanding the early evolutionary history of Archosauria. As the most recent and taxonomically comprehensive (at least regarding stem-archosaurs) phylogenetic analysis favours the originally proposed relationship of phytosaurs as members of Pseudosuchia (Ezcurra 2016), the alternative disparity patterns of non-archosaurian archosauromorphs and archosaurs are presented in the main text. Nevertheless, as highlighted in the main text, the evolutionary patterns

described and discussed there are still valid for the paraphyletic group composed of non-archosaurian archosauromorphs to the exclusion of phytosaurs.

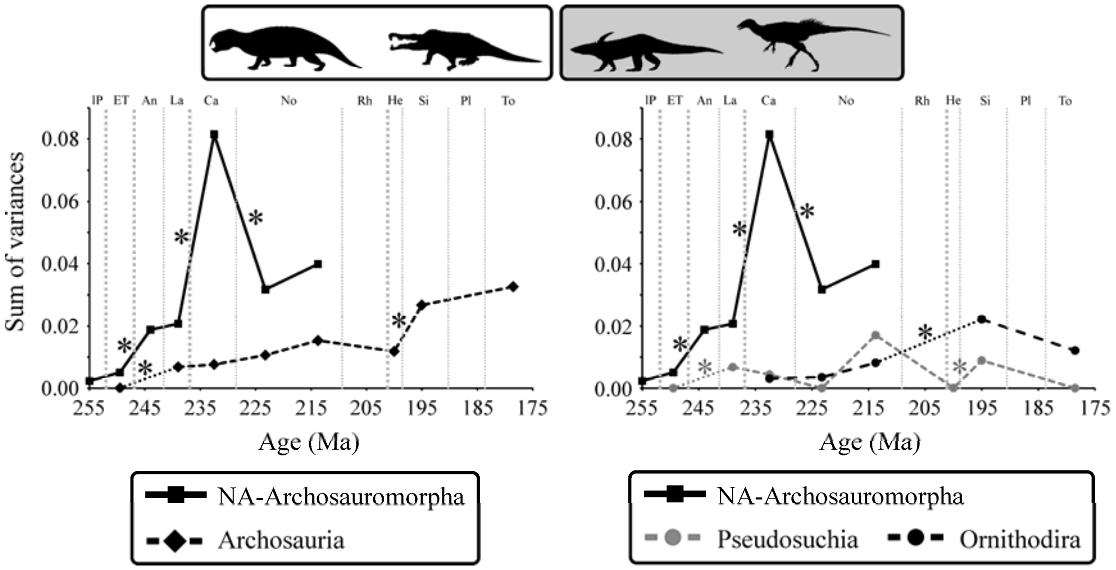

Figure S3. Disparity of archosauromorphs through time from the late Permian to the end of the Early Jurassic with phytosaurs as members of the stem group. Left: Temporal pattern of non-archosaurian archosauromorphs (solid line with black squares) and crown archosaurs (dashed line with black diamonds). Right: Temporal pattern showing non-archosaurian archosauromorphs (solid line with black squares) against pseudosuchians (dashed line with grey circles) and ornithodirans (dashed line with black circles). Significant changes between subsequent time bins are marked with an asterisk. Dotted lines mark missing time bins due to small sample sizes.

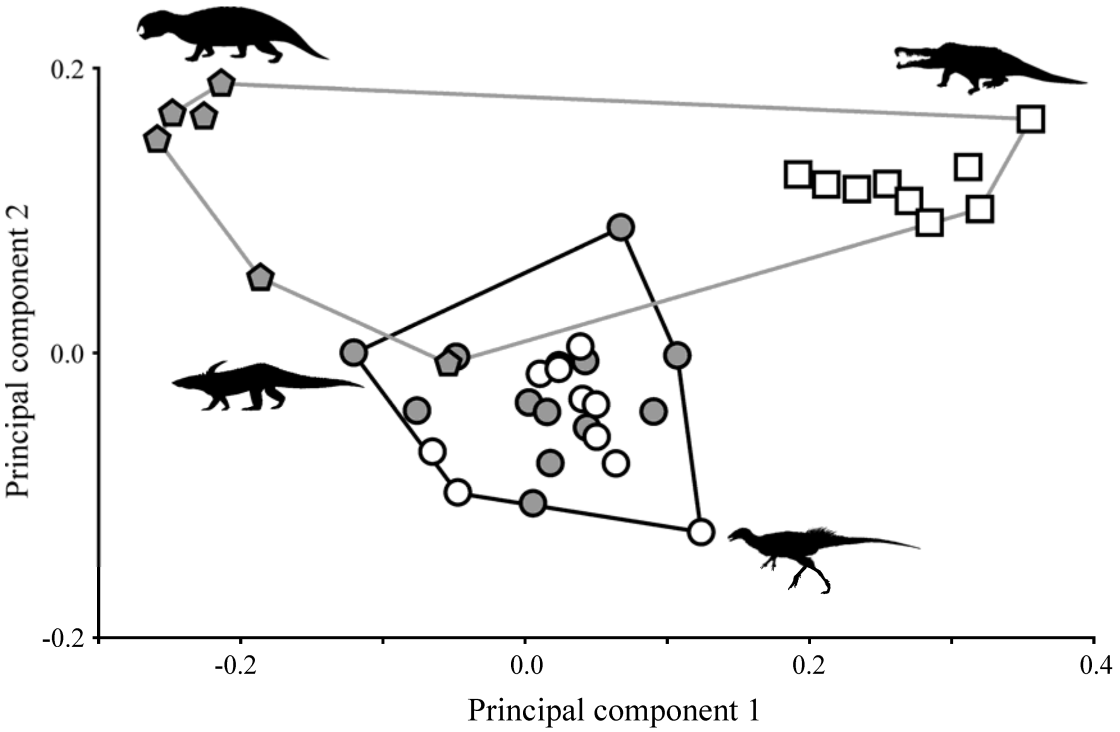

Figure S4. Two-dimensional morphospace of Late Triassic archosaurmorphs showing the morphospace of non-archosaurian archosauromorphs (grey solid line with grey pentagons) including phytosaurs (white squares) and crown archosaurs (black solid line) with pseudosuchians (grey circles) and ornithodirans (white circles).

5.3. Cranial disparity trends within Ornithodira

Due to their poor record in earlier time bins, the oldest time interval for which we can estimate disparity for ornithodirans is the Carnian. From that interval, cranial disparity increases continuously until the Sinemurian, with both Ornithodira and Dinosauria showing significant shifts in morphospace from the Carnian to the early Norian (Fig. 4, S5). Disparity changes from the late Norian to Hettangian could not be estimated due to poor sampling in the later bin, but a significant increase is observed from the late Norian to the Sinemurian. In the Toarcian, cranial disparity decreases again (Table S15). When compared to each other, Pseudosuchia (including phytosaurs) and Ornithodira occupy significantly different areas in morphospace over the entire time

span (Table S16). When phytosaurs are excluded from crown-group archosaurs, however, the morphospace of pseudosuchians and ornithodirans overlaps in the early Norian (Table S17). The general differences in cranial morphospace over time between ornithodirans and pseudosuchians suggests that the two groups probably did not compete extensively with each other, e.g. for similar food resources. However, due to the poor fossil record of ornithodirans prior to the Late Triassic (Langer et al. 2013), nothing can be said about potential ecological overlap in previous time periods.

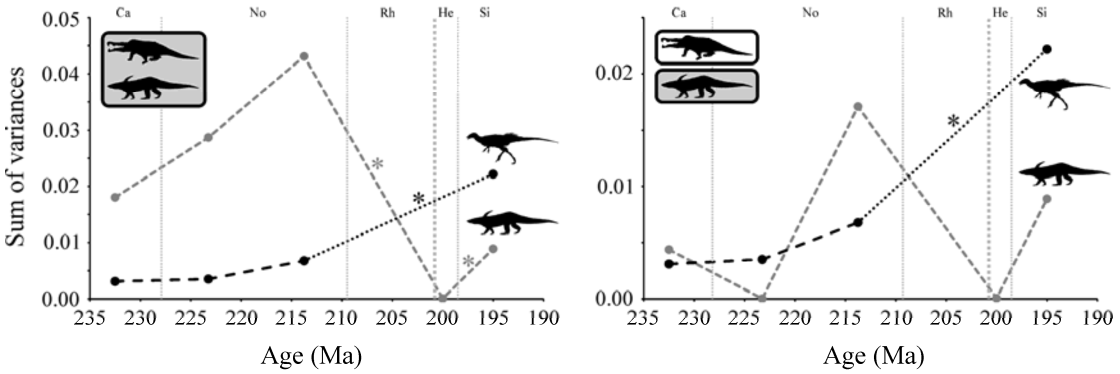

Figure S5. Temporal disparity of pseudosuchians (grey dashed line) and dinosaurs (black dashed line) from the Carnian to the end of the Early Jurassic. The left diagram shows the disparity trend when phytosaurs are members of the Pseudosuchia, while the right one shows the situation when phytosaurs are members of the stem-line.

5.4. The impact of ancestral shapes for disparity analyses

To study disparity changes through time we included the shapes of hypothetical ancestors to increase temporal resolution. To estimate the impact of the ancestors, we calculated the temporal disparity curves for all groups (when phytosaurs are members of crown Archosauria) on the basis of terminal taxa only and compared them with the results presented in the main text (Table S18-S23). Using only terminal taxa results in

slightly higher disparity. However, as the magnitude of sum of variance, the disparity metric used in this study, depends on the sample size, the higher disparity values are a mathematical artefact as the sample size is reduced due to the exclusion of ancestral shapes. The shapes of the disparity curves through time are generally similar for both approaches (Fig. S6), so that we assume that the basic trends described in this study are still valid. The inclusion of ancestral shapes furthermore allowed us to estimate disparity for earlier time bins and for those with low sample sizes. This is especially true for the Early Jurassic, where the fossil record of pseudosuchians and ornithodirans is generally low, complicating the estimation of statistically meaningful disparity values (Fig. S6).

Table S18. Sum of variances of Archosauromorpha (just terminal taxa) through time when phytosaurs are members of the crown. NA = non-archosaurian.

	**all Archosauromorpha**	**Ornithodira**	**Dinosauria**
**EARLY TRIASSIC (ET)**	0.0069	-	-
**ANISIAN (ANI)**	0.0246	-	-
**LADINIAN (LAD)**	0.0176	-	-
**CARNIAN (CAR)**	0.0527	0.0090	0.0090
**NORIAN1 (NOR1)**	0.0564	-	-
**NORIAN2 (NOR2)**	0.0369	0.0094	0.0081
**SINEMURIAN (SIN)**	0.0324	0.0288	0.0288
**TOARCIAN (TOA)**	0.0397	0.0210	-
**Phytosaurs as crown group**	**NA-Archosauromorpha**	**Archosauria**	**Pseudosuchia**
**EARLY TRIASSIC (ET)**	0.0069	-	-
**ANISIAN (ANI)**	0.0215	-	-
**LADINIAN (LAD)**	0.0237	0.0129	0.0129
**CARNIAN (CAR)**	0.0150	0.0240	0.0252
**NORIAN1 (NOR1)**	-	0.0346	0.0319
**NORIAN2 (NOR2)**	-	0.0351	0.0424
**SINEMURIAN (SIN)**	-	0.0324	-
**TOARCIAN (TOA)**	-	0.0397	-

Table S19. Statistical differences of sum of variances and morphospace position between subsequent time bins for all Archosauromorpha (just terminal taxa). Bold p-values represent significant results.

	**Sum of Variance**		**NPMANOVA**	
	**Difference**	**p-value**	**F**	**p-value**
**ET-ANI**	0.0177	**0.0047**	4.1770	0.5796
**ANI-LAD**	-0.0070	0.2463	1.5380	1.0000
**LAD-CAR**	0.0352	0.2013	0.5349	1.0000
**CAR-NOR1**	0.0036	0.8671	1.4830	1.0000
**NOR1-NOR2**	-0.0194	0.2061	0.8348	1.0000
**NOR2- SIN**	-0.0046	0.7739	3.1540	0.8148
**SIN-TOA**	0.0074	0.7641	2.3690	1.0000

Table S20. Statistical differences of sum of variances and morphospace position between subsequent time bins for non-archosaurian Archosauromorpha (just terminal taxa) when phytosaurs are members of the crown. Bold p-values represent significant results.

	**Sum of Variance**		**NPMANOVA**	
**Phytosauria as crown**	**Difference**	**p-value**	**F**	**p-value**
**ET-ANI**	0.0146	**0.0098**	5.8550	**0.0468**
**ANI-LAD**	0.0021	0.8186	2.0420	0.7086
**LAD-CAR**	-0.0086	0.8475	9.0920	0.6312

Table S21. Statistical differences of sum of variances and morphospace position between subsequent time bins for Archosauria (just terminal taxa) when phytosaurs are members of the crown. Bold p-values represent significant results.

	**Sum of Variance**		**NPMANOVA**	
**Phytosauria as crown**	**Difference**	**p-value**	**F**	**p-value**
**LAD-CAR**	0.0112	0.9163	1.1110	1.0000
**CAR-NOR1**	0.0106	0.4394	2.1960	1.0000
**NOR1-NOR2**	0.0005	0.9772	0.8255	1.0000
**NOR2- SIN**	-0.0028	0.8651	3.9240	0.2250
**SIN-TOA**	0.0074	0.7826	2.3690	0.9720

Table S22. Statistical differences of sum of variances and morphospace position between subsequent time bins for Pseudosuchia (just terminal taxa) when phytosaurs are members of the crown. Bold p-values represent significant results.

	**Sum of Variance**		**NPMANOVA**	
**Phytosauria as crown**	**Difference**	**p-value**	**F**	**p-value**
**LAD-CAR**	0.0123	0.9812	2.0760	0.5316
**CAR-NOR1**	0.0067	0.5331	1.6940	1.0000
**NOR1-NOR2**	0.0105	0.4711	0.9249	1.0000

Table S23. Statistical differences of sum of variances and morphospace position between subsequent time bins for Ornithodira/Dinosauria (just terminal taxa) when phytosaurs are members of the crown. Bold p-values represent significant results.

	**Sum of Variance**		**NPMANOVA**	
**Ornithodira**	**Difference**	**p-value**	**F**	**p-value**
**CAR-NOR2**	0.0004	0.9543	2.1970	0.5634
**NOR2-SIN**	0.0194	0.1162	2.0490	0.7866
**SIN-TOA**	-0.0079	0.5706	0.9408	1.0000
**Dinosauria**				
CAR-NOR2	-0.0009	0.9643	2.5320	0.3273
NOR2-SIN	0.0207	0.1229	2.0490	0.3585

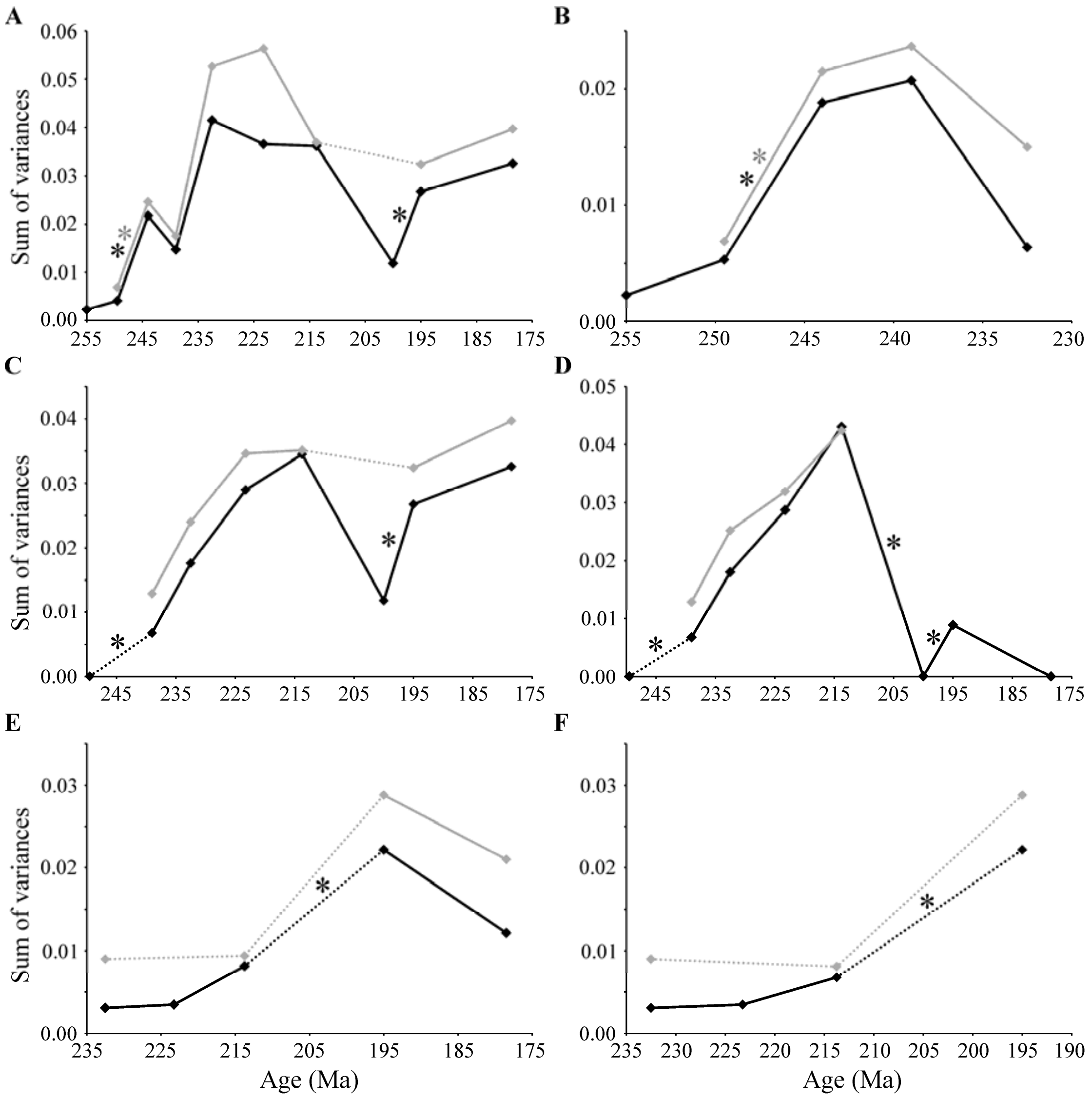

Figure S6. Comparison of disparity through time for various archosauromorph groups (when phytosaurs are crown archosaurs), if shapes of hypothetical ancestors were included (black line) or excluded (grey line). **(A)** Archosauromorpha. **(B)** Non-archosaurian Archosauromorpha. **(C)** Archosauria. **(D)** Pseudosuchia. **(E)** Ornithodira. **(F)** Dinosauria. Significant changes between subsequent time bins are marked with an asterisk. Dotted lines mark missing time bins due to small samples sizes.

5.5. The impact sample size for disparity analyses

To test the impact of sample size on the Sum of Variance disparity values recovered for each bin we performed Spearman's rank-order correlation tests and ordinary least squares (OLS) and generalized least squares (GLS) regression analyses with a first-order autoregressive model. Spearman's rank-order correlation test is a linear correlation test based on ranks, in which the r_s coefficient describes the strength of the correlation. This coefficient ranges from -1 to 1, in which $r_s = 0$ indicates no correlation (Hammer & Harper 2006). This test was performed in PAST (Hammer et al. 2001). While OLS estimates the strength of serial correlation between two variables by minimizing the sum of squares from a 'random' error (Hammer & Harper 2006), GLS uses maximum likelihood to correct for non-independence between adjacent points (Hansen 2007). Strength of correlation is given in form of R^2 for OLS and φ (dispersion parameter) and a correlation parameter for GLS. P-values indicate how significantly different the estimate slopes are from zero. All regression analyses were performed in R (R Development Core Team 2011), in which GLS was performed using the *gls()* function of the package 'nlme' (Pinheiro et al. 2016).

The majority of disparity curves show a weak correlation with sample size in the GLS regressions, but these correlations are significant only for all Archosauromorpha and Pseudosuchia (when phytosaurs are member of the crown). The latter result is also supported as significant by OLS and Spearman's correlation tests, while all other comparisons were non-significant. The correlations found for Archosauromorpha and Pseudosuchia might indicate that changes in disparity could be partly driven by sample size, and thus representing an artefact of variation in fossil record sampling through time. On the other hand, it could alternatively indicate that for these groups cranial disparity and species diversity are linked with each other,

indicating weak constraints on cranial disparity (see Wesley-Hunt 2005). Further discussion of this point is provided in the main text.

Table S24. Results of ordinary least squares (OLS) and generalised least squares (GLS) regressions, and Spearman's correlation of skull shape disparity and sample size per bin. Values in brackets show the p-value of the slope, in which bold values indicate a significant result.

	OLS		GLS			Spearman's
	Slope	R^2	Slope	φ	Correlation	r_s
**all Archosauromorpha**	324.0 (0.097)	0.3059	**294.3 (0.010)**	-0.1579	-0.8800	0.596 (0.069)
**Ornithodira**	28.1 (0.885)	0.0082	30.9 (0.877)	0.0502	-0.7960	-0.026 (0.933)
**Dinosauria**	56.2 (0.754)	0.0606	184.3 (0.068)	-0.8980	-0.8260	0.5 (0.667)
**Phytosaurs as crown group**						
**NA-Archosauromorpha**	-25.6 (0.922)	0.0037	-27.3 (0.919)	0.4133	-0.6150	-0.500 (0.450)
**Archosauria**	410.0 (0.119)	0.3555	410.7 (0.119)	0.0051	-0.8720	0.664 (0.093)
**Pseudosuchia**	**292.2 (0.001)**	0.8529	**294.3 (0.000)**	-0.4280	-0.7870	**0.915 (0.004)**
**Phytosaurs as stem group**						
**NA-Archosauromorpha**	-16.8 (0.761)	0.0202	-20.9 (0.702)	-0.0383	-0.7730	0.018 (0.987)
**Archosauria**	80.2 (0.732)	0.0210	46.9 (0.783)	-0.3834	-0.8410	0.323 (0.433)
**Pseudosuchia**	272.4 (0.088)	0.4089	261.5 (0.117)	-0.1276	-0.7060	0.639 (0.088)

5.6. The age of the Chañares Formation

During the writing of this manuscript, Marsicano et al. (2015) published the first radioisotopic dates for the Argentinean Chañares Formation (Ischigualasto-Villa Unión Basin). This unit was historically considered to be Ladinian (Rogers et al., 2001) or Ladinian−earliest Carnian in age (Desojo et al., 2011) based on biostratigraphical evidence and absolute dating of overlying formations. The dates published by Marsicano et al. (2015) are based on chemical abrasion thermal ionization mass spectrometry (CA-TIMS) of U−Pb zircons from two different stratigraphic layers that contain tuff in the lower member of the formation. These

dates (236.1±0.6 Ma and 233.7±0.4 Ma, respectively) indicate that most of the formation is early Carnian and not Ladinian, but since the Ladinian-Carnian boundary occurs at ca. 237 Ma (Gradstein et al., 2012) the lowermost portion of the unit could still be uppermost Ladinian in age. This new date for the Chañares Formation has an impact on the binning of three taxa used in our study: *Gracilisuchus stipanicicorum*, *Chanaresuchus bonapartei* and *Gualosuchus reigi*. These three species occur within the first 7–30 metres of the unit and thus are positioned very close to the Ladinian−Carnian boundary (MDE pers. obs.). As a result, the Ladinian−earliest Carnian age used here for these taxa is still valid.

6. References

Adams, D. C. 2014. A generalized K statistic for estimating phylogenetic signal from shape and other high-dimensional multivariate data. Syst. Biol. 63:685–697.

Adams, D. C., and E. Otárola-Castillo. 2013. geomorph: an R package for the collection and analysis of geometricmorphometric shape data. Methods Ecol. Evol. 4:393–399.

Andres, B., and T. S. Myers. 2013. Lone star pterosaurs. Earth Environ. Sci. Trans. R. Soc. Edinburgh 103:383–398.

Barrett, P. M., R. J. Butler, and F. Knoll. 2005. Small-bodied ornithischian dinosaurs from the Middle Jurassic of Sichuan, China. J. Vertebr. Paleontol. 25:823–834.

Bennett, S. C. 2014. A new specimen of the pterosaur *Scaphognathus crassirostris*, with comments on constraint of cervical vertebrae number in pterosaurs. Neues Jahrb. für Geol. und Paläontologie, Abhandlungen 271:327–348.

Benton, M. J. 1990. The species of *Rhynchosaurus*, a rhynchosaur (Reptilia, Diapsida) from the Middle Triassic of England. Philos. Trans. R. Soc. London, Ser. B 328:213–306.

Benton, M. J. 1983. The Triassic *Hyperodapedon* from Elgin: functional morphology and relationships. Philos. Trans. R. Soc. London, Ser. B 302:605–718.

Blomberg, S. P., T. J. Garland, and A. R. Ives. 2003. Testing for phylogenetic signal in comparative data: behavioral traits are more labile. Evolution (N. Y). 57:717–745.

Brill, K., and K. Carpenter. 2006. A description of a new ornithopod from the Lytle Member of the Purgatoire Formation (Lower Cretaceous) and a reassessment of the skull of *Camptosaurus*. Pp. 49–67 in K. Carpenter, ed. Horns and beaks: ceratopsian and ornithopod dinosaurs. Indiana University Press, Bloomington.

Bronzati, M., F. C. Montefeltro, and M. C. Langer. 2012. A species-level supertree of Crocodyliformes. Hist. Biol. 24:598–606.

Brusatte, S. L., M. J. Benton, J. B. Desojo, and M. C. Langer. 2010. The higher-level phylogeny of Archosauria (Tetrapoda: Diapsida). J. Syst. Palaeontol. 8:3–47.

Brusatte, S. L., M. J. Benton, M. Ruta, and G. T. Lloyd. 2008. Superiority, competition, and opportunism in the evolutionary radiation of dinosaurs. Science 321:1485–1488.

Butler, R. J., P. M. Galton, L. B. Porro, L. M. Chiappe, M. D. Henderson, and G. M. Erickson. 2010. Lower limits of ornithischian dinosaur body size inferred from a new Upper Jurassic heterodontosaurid from North America. Proc. R. Soc. B 277:375–381.

Butler, R. J., R. B. Irmis, and M. C. Langer. 2011. Late Triassic terrestrial biotas and the rise of dinosaurs. Earth Environ. Sci. Trans. R. Soc. Edinburgh 101:1–426.

Butler, R. J., C. Sullivan, M. D. Ezcurra, J. Liu, A. Lecuona, and R. B. Sookias. 2014. New clade of enigmatic early archosaurs yields insights into early pseudosuchian phylogeny and the biogeography of the archosaur radiation. BMC Evol. Biol. 14:128.

Cabreira, S. F., C. L. Schultz, J. S. Bittencourt, M. B. Soares, D. C. Fortier, L. R. Silva, and M. C. Langer. 2011. New stem-sauropodomorph (Dinosauria, Saurischia) from the Triassic of Brazil. Naturwissenschaften 98:1035–1040.

Camp, C. L. 1930. A study of the phytosaurs with description of new material from western North America. Mem. Univ. Calif. 10:1–161.

Carrano, M. T., R. B. J. Benson, and S. D. Sampson. 2012. The phylogeny of Tetanurae (Dinosauria: Theropoda). J. Syst. Palaeontol. 10:211–300.

Carvalho, I. S., V. P. A. Teixeira, M. L. F. Ferraz, L. C. B. Ribeiro, A. G. Martinelli, F. M. Neto, J. J. W. Sertich, G. C. Cunha, I. C. Cunha, and P. F. Farraz. 2011. *Campinasuchus dinizi* gen. et sp. nov., a new Late Cretaceous baurusuchid (Crocodyliformes) from the Bauru Basin, Brazil. Zootaxa 2871:19–42.

Carvalho, I. S., F. M. Vasconcellos, and S. A. S. Tavares. 2007. *Montealtosuchus arrudacamposi*, a new peirosaurid crocodile (Mesoeucrocodylia) from the Late Cretaceous Adamantina Formation of Brazil. Zootaxa 1607:35–46.

Case, E. C. 1929. Description of the skull of a new form of phytosaur with notes on the characters of described North American phytosaurs. Mem. Univ. Michigan Museums 2:1–56.

Case, E. C., and T. E. White. 1934. Two new specimens of phytosaurs from the Upper Triassic of western Texas. Contrib. from Museum Paleontol. 4:133–142.

Chatterjee, S. 1974. A Rhynchosaur from the upper Triassic Maleri Formation of india. Philos. Trans. R. Soc. London, Ser. B 267:209–261.

Cubo, J., N. Le Roy, C. Martinez-Maza, and L. Montes. 2012. Paleohistological estimation of bone growth rate in extinct archosaurs. Paleobiology 38:335–349.

Currie, P. J., and X. Zhao. 1993. A new carnosaur (Dinosauria, Theropoda) from the Jurassic of Xinjiang, People's Republic of China. Can. J. Earth Sci. 30:2037–2081.

Desojo, J. B., and A. M. Báez. 2007. Cranial morphology of the Late Triassic South American archosaur *Neoaetosauroides engaeus*: evidence for aetosaurian diversity. Palaeontology 50:267–276.

Desojo, J. B., M. D. Ezcurra, and E. E. Kischlat. 2012. A new aetosaur genus (Archosauria: Pseudosuchia) from the early Late Triassic of southern Brazil. Zootaxa 3166:1–33.

Desojo, J. B., M. D. Ezcurra, and C. L. Schultz. 2011. An unusual new archosauriform from the Middle-Late Triassic of southern Brazil and the monophyly of Doswelliidae. Zool. J. Linn. Soc. 161:839–871.

Dilkes, D. 1998. The Early Triassic rhynchosaur *Mesosuchus browni* and the interrelationships of basal archosauromorph reptiles. Philos. Trans. R. Soc. London, Ser. B 353:501–541.

Dilkes, D., and A. B. Arcucci. 2012. *Proterochampsa barrionuevoi* (Archosauriformes: Proterochampsia) from the Late Triassic (Carnian) of Argentina and a phylogenetic analysis of Proterochampsia. Palaeontology 55:853–885.

Ezcurra, M. D. 2007. The cranial anatomy of the coelophysoid theropod *Zupaysaurus rougieri* from the Upper Triassic of Argentina. Hist. Biol. 19:185–202.

Ezcurra, M. D. 2016. The phylogenetic relationships of basal archosauromorphs, with an emphasis on the systematics of proterosuchian archosauriforms. PeerJ.

Ezcurra, M. D., and R. J. Butler. 2015a. Post-hatchling cranial ontogeny in the Early Triassic diapsid reptile *Proterosuchus fergusi*. J. Anat. 226:387–402.

Ezcurra, M. D., and R. J. Butler. 2015b. Taxonomy of the proterosuchid archosauriforms (Diapsida: Archosauromorpha) from the earliest Triassic of South Africa, and implications for the early archosauriform radiation. Palaeontology 58:141–170.

Ezcurra, M. D., T. M. Scheyer, and R. J. Butler. 2014. The origin and early evolution of Sauria: reassessing the Permian saurian fossil record and the timing of the crocodile-lizard divergence. PLoS One 9:e89165.

Fiorelli, L., and J. O. Calvo. 2008. New remains of *Notosuchus terrestris* Woodward, 1896 (Crocodyliformes: Mesoeucrocodylia) from Late Cretaceous of Neuquén, Patagonia, Argentina. Arq. do Mus. Nac. Rio Janeiro 66:83–124.

Flynn, J., S. J. Nesbitt, J. M. Parrish, L. Ranivoharimanana, and A. R. Wyss. 2010. A new species of *Azendohsaurus* (Diapsida: Archosauromorpha) from the Triassic Isalo Group of southwestern Madagascar: cranium and mandible. Palaeontology 53:669–688.

Foth, C., H. Tischlinger, and O. W. M. Rauhut. 2014. New specimen of *Archaeopteryx* provides insights into the evolution of pennaceous feathers. Nature 511:79–82.

Gomani, E. M. 1997. A crocodyliform from the Early Cretaceous dinosaur beds, Northern Malawi. J. Vertebr. Paleontol. 17:280–294.

Gottmann-Quesada, A., and P. M. Sander. 2009. A redescription of the early archosauromorph *Protorosaurus speneri* Meyer, 1832 and its phylogenetic relationships. Palaeontogr. Abt. A 287:123–220.

Gow, C. E., J. W. Kitching, and M. A. Raath. 1990. Skulls of the prosauropod dinosaur *Massospondylus carinatus* Owen in the collections of the Bernand Price Institute for Palaeontological Research. Palaeontol. Africana 27:45–58.

Gower, D. J., and A. G. Sennikov. 1996. Morphology and phylogenetic informativeness of early archosaur braincases. Palaeontology 39:883–906.

Gradstein, F. M., J. G. Ogg, M. D. Schmitz, and G. M. Ogg. 2012. The Geologic Time Scale 2012. Elsevier Academic Press, Amsterdam.

Hammer, O. and Harper, D. A. T. 2006. Paleontological data analysis. Blackwell Publishing, Malden.

Hammer O, Harper DAT, Ryan PD. 2001. PAST: paleontological statistics software package for education and data analysis. Palaeontol. Electron. 4:1–9.

Hansen, C. B. 2007. Generalized least squares inference in panel and multilevel models with serial correlation and fixed effects. J. Econometrics 140:670–694.

Haubold, H. 1990. Ein neuer Dinosaurier (Ornithischia, Thyreophora) aus dem Unteren Jura des nördlichen Mitteleuropa. Rev. Paléobiologie 9:149–177.

Hone, D. W. E., and M. J. Benton. 2008. A new genus of rhynchosaur from the Middle Triassic of south-west England. Palaeontology 51:95–115.

Hübner, T. R., and O. W. M. Rauhut. 2010. A juvenile skull of *Dysalotosaurus lettowvorbecki* (Ornithischia: Iguanodontia), and implications for cranial ontogeny, phylogeny, and taxonomy in ornithopod dinosaurs. Zool. J. Linn. Soc. 160:366–396.

Hungerbühler, A. 2002. The Late Triassic phytosaur *Mystriosuchus westphali*, with a revision of the genus. Palaeontology 45:377–418.

Irmis, R. B., and J. H. Whiteside. 2012. Delayed recovery of non-marine tetrapods after the end-Permian mass extinction tracks global carbon cycle. Proc. R. Soc. B 279:1310–1318.

Kley, N. J., J. J. W. Sertich, A. H. Turner, D. W. Krause, P. M. O'Connor, and J. A. Georgi. 2010. Craniofacial morphology of *Simosuchus clarki* (Crocodyliformes: Notosuchia) from the Late Cretaceous of Madagascar. J. Vertebr. Paleontol. 30:13–98.

Klingenberg, C. P. 2011. MorphoJ: an integrated software package for geometric morphometrics. Mol. Ecol. Resour. 11:353–357.

Klingenberg, C. P., and N. A. Gidaszewski. 2010. Testing and quantifying phylogenetic signals and homoplasy in morphometric data. Syst. Biol. 59:245–261.

Langer, M. C., F. C. Montefeltro, D. W. E. Hone, R. Whatley, and C. L. Schultz. 2010. On *Fodonyx spenceri* and a new rhynchosaur from the Middle Triassic of Devon. J. Vertebr. Paleontol. 30:1884–1888.

Langer, M. C., S. J. Nesbitt, J. S. Bittencourt, and R. B. Irmis. 2013. Non-dinosaurian Dinosauromorpha. Geol. Soc. London, Spec. Publ. 379:157–186.

Langer, M. C., and C. L. Schultz. 2000. A new species of the Late Triassic rhynchosaur *Hyperodapedon* from the Santa Maria Formation of south Brazil. Palaeontology 43:633–652.

Larsson, H. C. E., and H.-D. Sues. 2007. Cranial osteology and phylogenetic relationships of *Hamadasuchus rebouli* (Crocodyliformes: Mesoeucrocodylia) from the Cretaceous of Morocco. Zool. J. Linn. Soc. 149:533–567.

Lau, K. V, K. Maher, D. Altiner, B. M. Kelley, L. R. Kump, D. J. Lehrmann, J. C. Silva-Tamayo, K. L. Weaver, M. Yu, and J. L. Payne. 2016. Marine anoxia and

delayed Earth system recovery after the end-Permian extinction. Proc. Natl. Acad. Sci. 113:2360 – 2365.

Laurin, M. 2004. The evolution of body size, Cope's rule and the origin of amniotes. Syst. Biol. 53:594–622.

Leal, L. A., S. A. K. Azevedo, A. W. A. Kellner, and A. A. S. Da Rosa. 2004. A new early dinosaur (Sauropodomorpha) from the Caturrita Formation (Late Triassic), Paraná Basin, Brazil. Zootaxa 690:1–24.

Li, C., X. Wu, Y. Cheng, T. Sato, and L. Wang. 2006. An unusual archosaurian from the marine Triassic of China. Naturwissenschaften 93:200–206.

Loewen, M. A. 2009. Variation in the Late Jurassic theropod dinosaur *Allosaurus*: ontogenetic, functional, and taxonomic implications. University of Utah, Salt Lake City.

Maidment, S. C. R., D. B. Norman, P. M. Barrett, and P. Upchurch. 2008. Systematics and phylogeny of Stegosauria (Dinosauria: Ornithischia). J. Syst. Palaeontol. 6:1815–1821.

Mannion, P. D., R. B. J. Benson, M. T. Carrano, J. P. Tennant, J. Judd, and R. J. Butler. 2015. Climate constrains the evolutionary history and biodiversity of crocodylians. Nat. Commun. 6:8438.

Marsicano, C. A., R. B. Irmis, A. C. Mancuso, R. Mundil, and F. Chemale. 2015. The precise temporal calibration of dinosaur origins. Proc. Natl. Acad. Sci. 113:509–513.

Martínez, R. N. 2009. *Adeopapposaurus mognai*, gen. et sp. nov. (Dinosauria: Sauropodomorpha), with comments on adaptations of basal Sauropodomorpha. J. Vertebr. Paleontol. 29:142–164.

Martínez, R. N., C. Apaldetti, and D. Abelin. 2013. Basal sauropodomorphs from the Ischigualasto Formation. Soc. Vertebr. Paleontol. Mem. 12:51–69.

McDonald, A. T., J. I. Kirkland, D. D. DeBlieux, S. K. Madsen, J. Cavin, A. R. Milner, and L. Panzarin. 2010. New basal iguanodonts from the Cedar Mountain Formation of Utah and the evolution of thumb-spiked dinosaurs. PLoS One 5:e14075.

Montefeltro, F. C., M. C. Langer, and C. L. Schultz. 2010. Cranial anatomy of a new genus of hyperodapedontine rhynchosaur (Diapsida, Archosauromorpha) from the Upper Triassic of southern Brazil. Earth Environ. Sci. Trans. R. Soc. Edinburgh 101:27–52.

Nesbitt, S. J. 2011. The early evolution of archosaurs: relationships and the origin of major clades. Bull. Am. Museum Nat. Hist. 352:1–292.

Nesbitt, S. J., J. B. Desojo, and R. B. Irmis. 2013. Anatomy, phylogeny and palaeobiology of early archosaurs and their kin. Geol. Soc. London, Spec. Publ. 379:1–608. London.

Norman, D. B., A. W. Crompton, R. J. Butler, L. B. Porro, and A. J. Charig. 2011. The Lower Jurassic ornithischian dinosaur *Heterodontosaurus tucki* Crompton & Charig, 1962: cranial anatomy, functional morphology, taxonomy, and relationships. Zool. J. Linn. Soc. 163:182–276.

Nosotti, S. 2007. *Tanystropheus longobardicus* (Reptilia, Protorosauria) : re-interpretations of the anatomy based on new specimens from the Middle Triassic of Besano (Lombardy, Northern Italy). Mem. della Soc. Ital. di Sci. Nat. e del Mus. Civ. di Stor. Nat. di Milano 35:1–88.

Osmólska, H., S. Hua, and E. Buffetaut. 1997. *Gobiosuchus kielanae* (Protosuchia) from the Late Cretaceous of Mongolia: anatomy and relationships. Acta Palaeontol. Pol. 42:257–289.

Ouyang, H., and Y. Ye. 2002. The first mamenchisaurian skeleton with complete skull, *Mamenchisaurus youngi*. Sichuan Science and Technology Press, Chengdu.

Paradis, E. 2012. Analysis of phylogenetics and evolution with R. Springer, New York.

Parker, W. G., and R. B. Irmis. 2006. A new species of the Late Triassic phytosaur *Pseudopalatus* (Archosauria: Pseudosuchia) from Petrified Forest National Park, Arizona. Museum North. Arizona Bull. 62:126–143.

Peyer, K. 2006. A reconsideration of *Compsognathus* from the Upper Tithonian of Canjuers, southeastern France. J. Vertebr. Paleontol. 26:879–896.

Pierce, S. E., K. D. Angielczyk, and E. J. Rayfield. 2009. Shape and mechanics in thalattosuchian (Crocodylomorpha) skulls: implications for feeding behaviour and niche partitioning. J. Anat. 215:555–576.

Pierce, S. E., and M. J. Benton. 2006. *Pelagosaurus typus* Bronn, 1841 (Mesoeucrocodylia: Thalattosuchia) from the Upper Lias (Toarcian, Lower Jurassic) of Somerset, England. J. Vertebr. Paleontol. 26:621–635.

Pinheiro, J., Bates, D., DebRoy, S., Sarkar, D., EISPACK, Heisterkamp, S., Van Willigen, B. and R Development Core Team. 2016. nlme: linear and nonlinear mixed effects models. R package version 3.1-126: 1–336.

Pol, D., and Z. Gasparini. 2009. Skull anatomy of *Dakosaurus andiniensis* (Thalattosuchia: Crocodylomorpha) and the phylogenetic position of Thalattosuchia. J. Syst. Palaeontol. 7:163–197.

Pol, D., P. M. Nascimento, A. B. Carvalho, C. Riccomini, R. A. Pires-Domingues, and H. Zaher. 2014. A new notosuchian from the Late Cretaceous of Brazil and the phylogeny of advanced notosuchians. PLoS One 9:e93105.

Pol, D., and O. W. M. Rauhut. 2012. A Middle Jurassic abelisaurid from Patagonia and the early diversification of theropod dinosaurs. Proc. R. Soc. B 279:3170–3175.

Pritchard, A. C., A. H. Turner, S. J. Nesbitt, R. B. Irmis, and N. D. Smith. 2015. Late Triassic tanystropheids (Reptilia, Archosauromorpha) from northern New Mexico (Petrified Forest Member, Chinle Formation) and the biogeography, functional morphology, and evolution of Tanystropheidae. J. Vertebr. Paleontol. 35:e911186.

Rauhut, O. W. M. 2014. New observations on the skull of *Archaeopteryx*. Paläontolologische Zeitschrift 88:211–221.

Rauhut, O. W. M. 2003. The interrelationships and evolution of basal theropod dinosaurs. Spec. Pap. Palaeontol. 69:1–213.

Rauhut, O. W. M., R. Fechner, K. Remes, and K. Reis. 2011. How to get big in the Mesozoic: the evolution of the sauropodomorph body plan. Pp. 119–149 in N. Klein, K. Remes, C. T. Gee, and P. M. Sander, eds. Biology of the sauropod dinosaurs: understanding the life of giants. Indiana University Press, Bloomington.

R Development Core Team. 2011. R: a language and environment for statistical computing. http://www.r-project.org. R Foundation for Statistical Computing, Vienna.

Rogers, R. R., A. B. Arcucci, F. Abdala, P. C. Sereno, C. A. Forster, and C. May. 2001. Paleoenvironment and taphonomy of the Chañares Formation tetrapod assemblage (Middle Triassic), northwestern Argentina: spectacular preservation in volcanogenic concretions. Palaios 16:461–481.

Romer, A. S. 1971. The Chanares (Argentina) Triassic reptile fauna. XI. Two new long-snouted thecodonts, *Chanaresuchus* and *Gualosuchus*. Breviora 379:1–22.

Salisbury, S. W., R. E. Molnar, E. Frey, and P. M. A. Willis. 2006. The origin of modern crocodyliforms: new evidence from the Cretaceous of Australia. Proc. R. Soc. B 273:2439–2448.

Saller, F., S. Renesto, and F. M. Dalla Vecchia. 2013. First record of *Langobardisaurus* (Diapsida, Protorosauria) from the Norian (Late Triassic) of Austria, and a revision of the genus. Neues Jahrb. für Geol. und Paläontologie, Abhandlungen 268:83–95.

Sampson, S. D., and L. M. Witmer. 2007. Craniofacial anatomy of *Majungasaurus crenatissimus* (Theropoda: Abelisauridae) from the Late Cretaceous of Madagascar. Soc. Vertebr. Paleontol. Mem. 8:32–102.

Schoch, R. R. 2007. Osteology of the small archosaur *Aetosaurus* from the Upper Triassic of Germany. Neues Jahrb. für Geol. und Paläontologie, Abhandlungen 246:1–35.

Sereno, P. C. 1991. Basal archosaurs: phylogenetic relationships and functional implications. Soc. Vertebr. Paleontol. Mem. 2:1–53.

Sereno, P. C., S. Chao, Z. Cheng, and C. Rao. 1988. *Psittacosaurus meileyingensis* (Ornithischia: Ceratopsia), a new psittacosaur from the Lower Cretaceous of northeastern China. J. Vertebr. Paleontol. 8:366–377.

Sereno, P. C., and Z. Dong. 1992. The skull of the basal stegosaur *Huayangosaurus taibaii* and a cladistic diagnosis of Stegosauria. J. Vertebr. Paleontol. 12:318–343.

Sereno, P. C., and H. C. E. Larsson. 2009. Cretaceous crocodyliforms from the Sahara. Zookeys 28:1–143.

Sereno, P. C., H. C. E. Larsson, C. A. Sidor, and B. Gado. 2001. The giant crocodyliform Sarcosuchus from the Cretaceous of Africa. Science 294:1516–1519.

Sereno, P. C., R. N. Martínez, and O. A. Alcober. 2013. Osteology of *Eoraptor lunensis* (Dinosauria, Sauropodomorpha). Soc. Vertebr. Paleontol. Mem. 12:83–179.

Sertich, J. J. W., and P. M. O'Connor. 2014. A new crocodyliform from the middle Cretaceous Galula Formation, southwestern Tanzania. J. Vertebr. Paleontol. 34:576–596.

Sidor, C. A., D. A. Vilhena, K. D. Angielczyk, A. K. Huttenlocker, S. J. Nesbitt, B. R. Peecook, J. S. Steyer, R. M. H. Smith, and L. A. Tsuji. 2013. Provincialization of terrestrial faunas following the end-Permian mass extinction. Proc. Natl. Acad. Sci. 110:8129–8133.

Small, B. J. 2002. Cranial anatomy of *Desmatosuchus haplocerus* (Reptilia: Archosauria: Stagonolepididae). Zool. J. Linn. Soc. 136:97–111.

Small, B. J., and J. W. Martz. 2013. A new aetosaur from the Upper Triassic Chinle Formation of the Eagle Basin, Colorado, USA. Geol. Soc. London, Spec. Publ. 379:393–412.

Sookias, R. B., R. B. J. Benson, and R. J. Butler. 2012a. Biology, not environment, drives major patterns in maximum tetrapod body size through time. Biol. Lett. 8:674–677.

Sookias, R. B., R. J. Butler, and R. B. J. Benson. 2012b. Rise of dinosaurs reveals major body-size transitions are driven by passive processes of trait evolution. Proc. R. Soc. B 279:2180–2187.

Stocker, M. R. 2010. A new taxon of phytosaur (Archosauria: Pseudosuchia) from the Late Triassic (Norian) Sonsela Member (Chinle Formation) in Arizona, and a critical reevaluation of Leptosuchus Case, 1922. Palaeontology 53:997–1022.

Sues, H.-D., and N. C. Fraser. 2010. Triassic life on land. Columbia University Press, New York.

Sues, H.-D., S. J. Nesbitt, D. S. Berman, and A. C. Henrici. 2011. A late-surviving basal theropod dinosaur from the latest Triassic of North America. Proc. R. Soc. B 278:3459–3464.

Sulej, T. 2010. The skull of an early Late Triassic aetosaur and the evolution of the stagonolepidid archosaurian reptiles. Zool. J. Linn. Soc. 158:860–881.

Toljagic, O., and R. J. Butler. 2013. Triassic/Jurassic mass extinction as trigger for the Mesozoic radiation of crocodylomorphs. Biol. Lett. 9:20130095.

Turner, A. H., and G. A. Buckley. 2008. *Mahajangasuchus insignis* (Crocodyliformes: Mesoeucrocodylia) cranial anatomy and new data on the origin of the eusuchian-style palate. J. Vertebr. Paleontol. 28:382–408.

Tykoski, R. S. 2005. Anatomy, ontogeny, and phylogeny of coelophysoid theropods. The University of Texas, Austin.

Wang, R., S. Xu, X. Wu, C. Li, and S. Wang. 2013. A new specimen of *Shanisuchus shanisuchus* Young, 1964 (Diapsida: Archosauriformes) from the Triassic of Shanxi, China. Acta Geol. Sin. 87:1185–1197.

Weinbaum, J. C. 2011. The skull of *Postosuchus kirkpatricki* (Archosauria: Paracrocodyliformes) from the Upper Triassic of the United States. PaleoBios 30:18–44.

Wellnhofer, P. 1975. Die Rhamphorhynchoidea (Pterosauria) der Oberjura-Plattenkalke Süddeutschlands. Palaeontogr. Abt. A 148:1–185.

Wellnhofer, P. 1978. Pterosauria. Gustav Fischer Verlag, Stuttgart, New York.

Wesley-Hunt GD. 2005. The morphological diversification of carnivores in North America. Paleobiology 31:35–55.

Westphal, F. 1976. Phytosauria. Pp. 99–120 in O. Kuhn, ed. Handbuch der Paläoherpetologie. Gustav Fischer Verlag, Stuttgart.

Wilson, J. A. 2002. Sauropod dinosaur phylogeny: critique and cladistic analysis. Zool. J. Linn. Soc. 136:217–276.

Wilson, J. A., and P. C. Sereno. 1998. Early evolution and higher-level phylogeny of sauropod dinosaurs. Soc. Vertebr. Paleontol. Mem. 5:1–68.

Wu, X., and S. Chatterjee. 1993. *Dibothrosuchus elaphros*, a crocodylomorph from the Lower Jurassic of China and the phylogeny of the Sphenosuchina. J. Vertebr. Paleontol. 13:58–89.

Wu, X., A. P. Russell, and D. B. Brinkman. 2001. A review of *Leidyosuchus canadensis* Lambe, 1907 (Archosauria: Crocodylia) and an assessment of cranial variation based upon new material. Can. J. Earth Sci. 38:1665–1687.

Xu, L., J. M. Clark, J. Mo, J. N. Choiniere, C. A. Forster, G. M. Erickson, D. W. E. Hone, C. Sullivan, D. A. Eberth, S. J. Nesbitt, Q. Zhao, R. Hernandez, C. Jia, F. Han, and Y. Guo. 2009. A Jurassic ceratosaur from China helps clarify avian digital homologies. Nature 459:940–944.

Xu, X., J. M. Clark, C. A. Forster, M. A. Norell, G. M. Erickson, D. A. Eberth, C. Jia, and Q. Zhao. 2006a. A basal tyrannosauroid dinosaur from the Late Jurassic of China. Nature 439:715–718.

Xu, X., C. A. Forster, J. M. Clark, and J. Mo. 2006b. A basal ceratopsian with transitional features from the Late Jurassic of northwestern China. Proc. R. Soc. B 273:2135–2140.

Yates, A. M. 2012. Basal Sauropodomorpha: the “prosauropods.” Pp. 425–444 in M. K. Brett-Surman, T. R. J. Holtz, and J. O. Farlow, eds. The complete dinosaur. Indiana University Press, Bloomington.

Yates, A. M. 2007. The first complete skull of the Triassic dinosaur *Melanorosaurus* Haughton (Sauropodomorpha: Anchisauria). Spec. Pap. Palaeontol. 77:9–55.

Yates, A. M. 2003. The species taxonomy of the sauropodomorph dinosaurs from the Löwenstein Formation (Norian, Late Triassic) of Germany. Palaeontology 46:317–337.

Young, M. T., S. L. Brusatte, M. Ruta, and M. B. Andrade. 2010. The evolution of Metriorhynchoidea (Mesoeucrocodylia, Thalattosuchia): an integrated approach using geometric morphometrics, analysis of disparity, and biomechanics. Zool. J. Linn. Soc. 158:801–859.

Zaher, H., D. Pol, A. B. Carvalho, C. Riccomini, D. Campos, and W. Nava. 2006. Redescription of the cranial morphology of *Mariliasuchus amarali*, and its phylogenetic affinities (Crocodyliformes, Notosuchia). Am. Museum Novit. 3512:1–40.
